# Supplementary figures and images for: STING Contributes to Cancer-Induced Bone Pain by Promoting M1 Polarization of Microglia in the Medial Prefrontal Cortex
Source: Cancers (Basel). 2022 Oct 22;14(21):5188. doi: 10.3390/cancers14215188 (PMC9656586; doi:10.3390/cancers14215188)

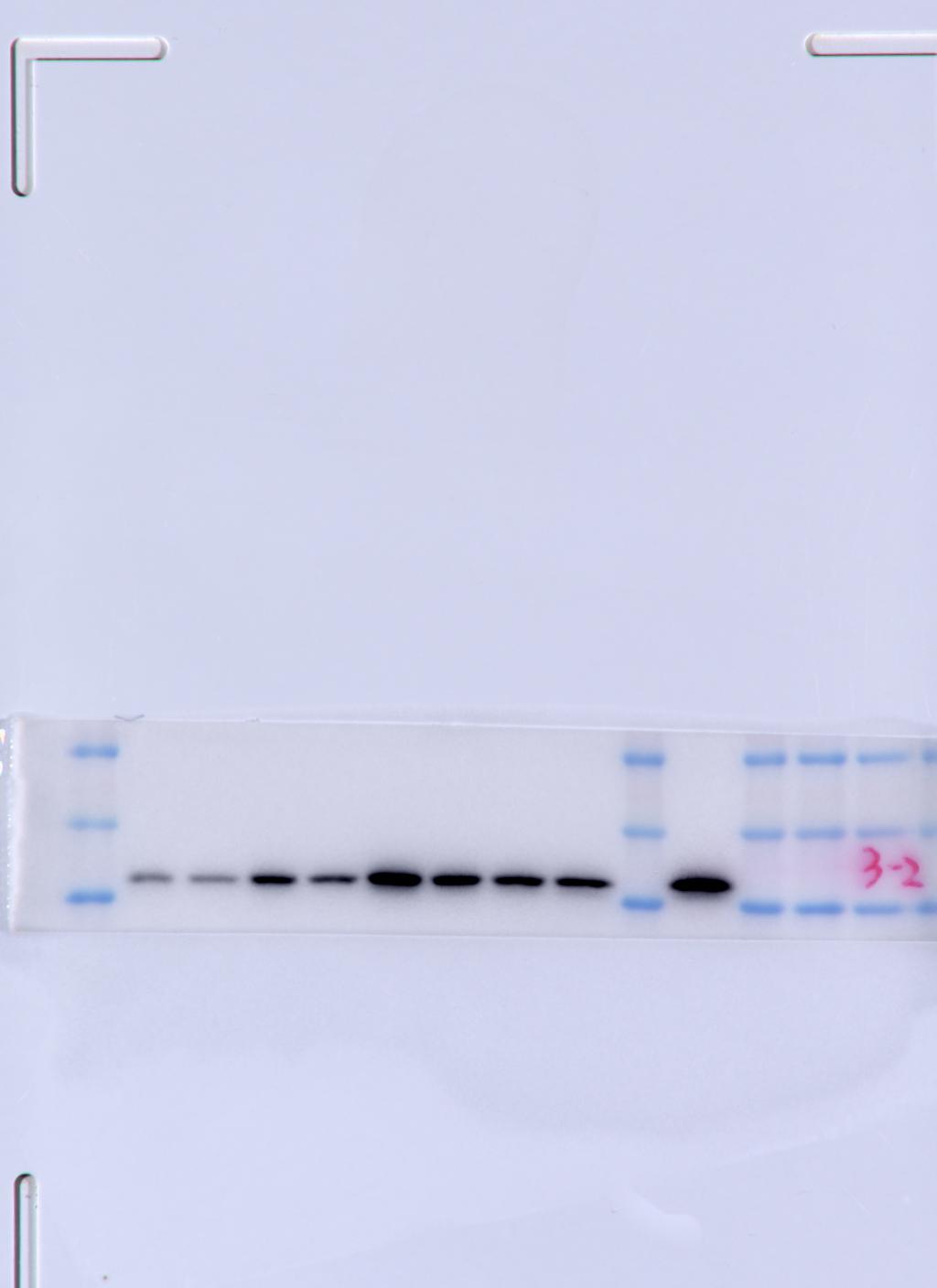

Supplement: Supplementary file 1 [file cancers-14-05188-s001.zip › WB(STING)/Figure 1E STING 2.jpg]

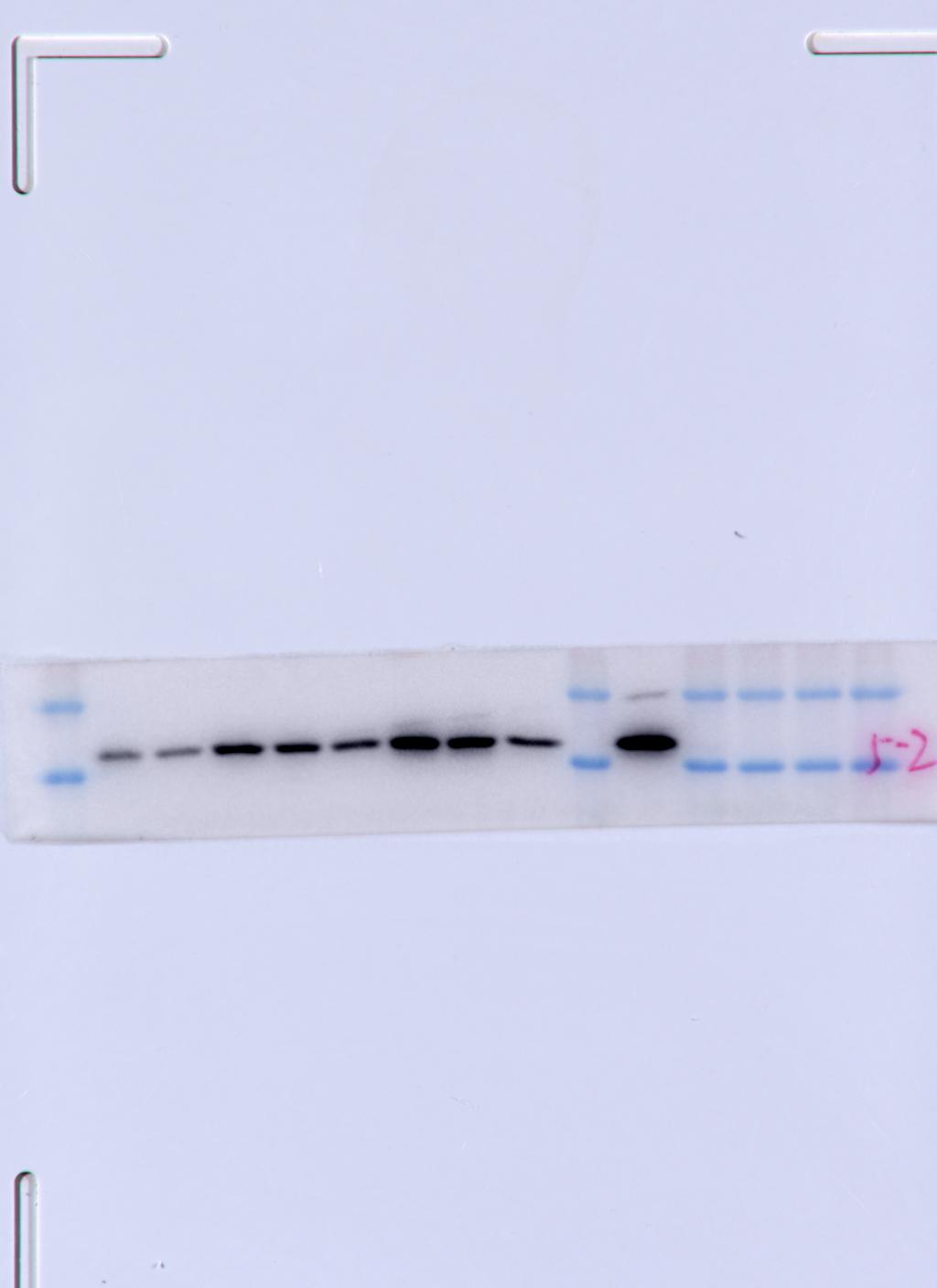

Supplement: Supplementary file 1 [file cancers-14-05188-s001.zip › WB(STING)/Figure 1E STING.jpg]

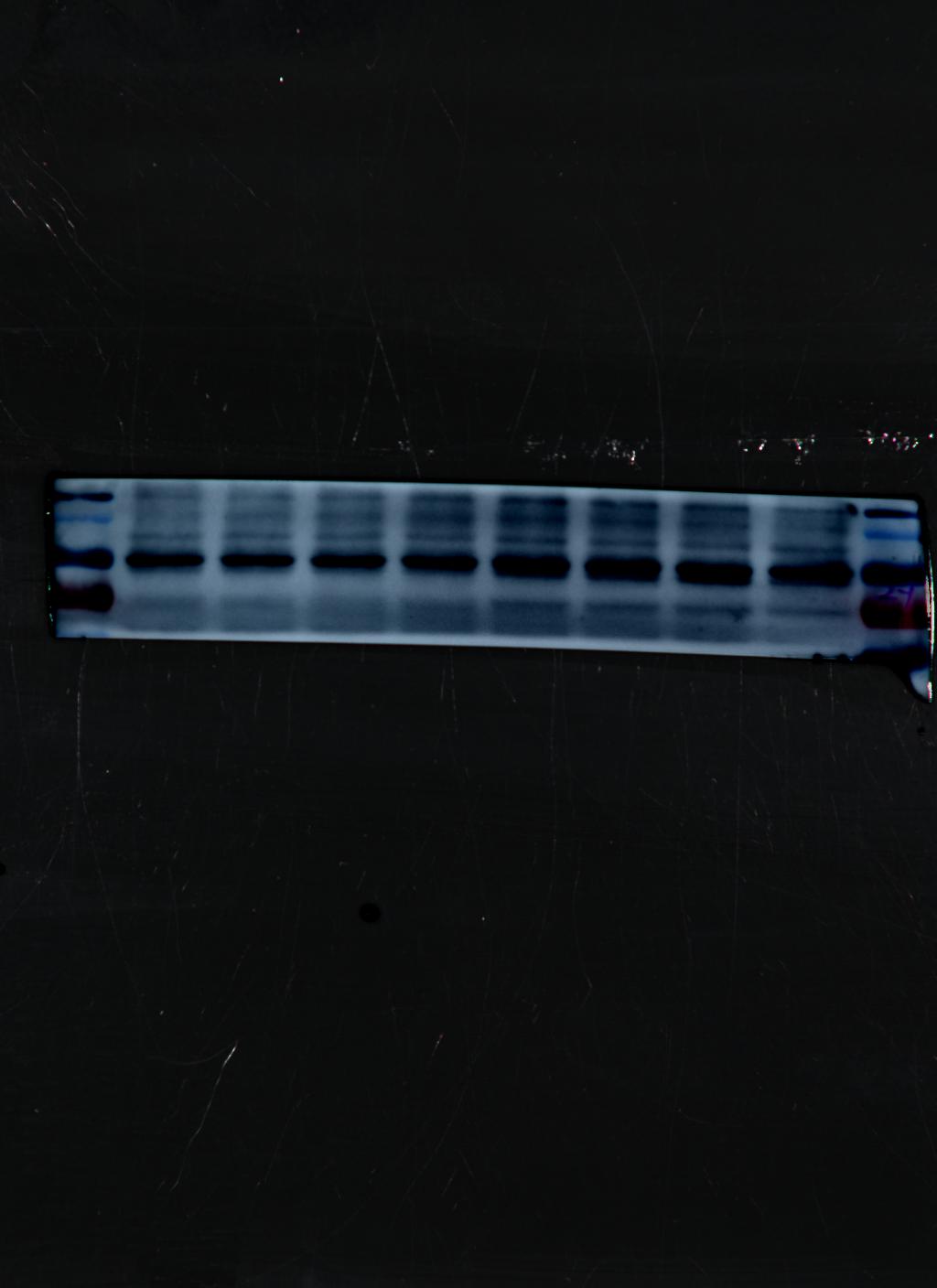

Supplement: Supplementary file 1 [file cancers-14-05188-s001.zip › WB(STING)/figure 2 p-TBK1.jpg]

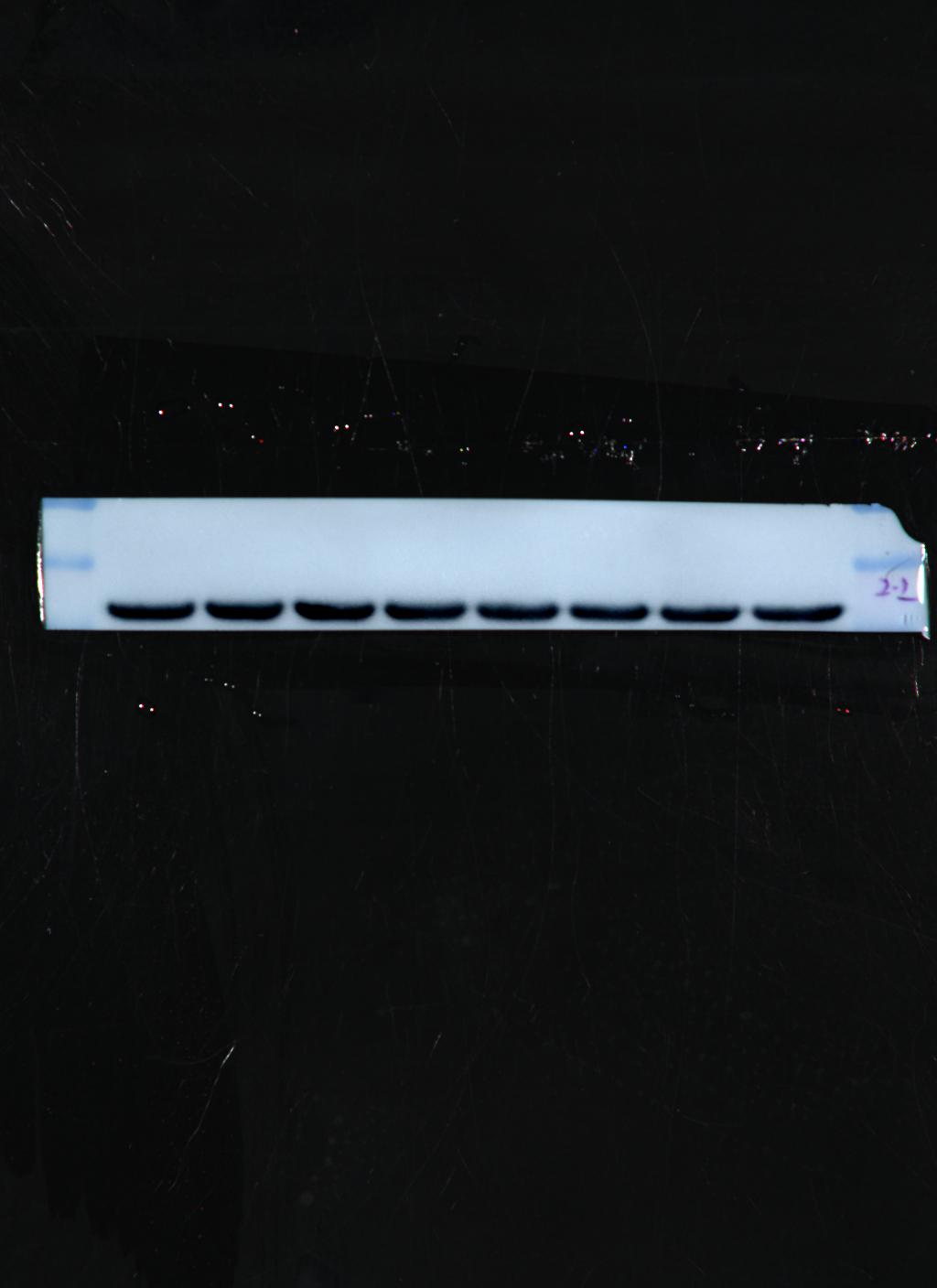

Supplement: Supplementary file 1 [file cancers-14-05188-s001.zip › WB(STING)/figure 2C gapdh.jpg]

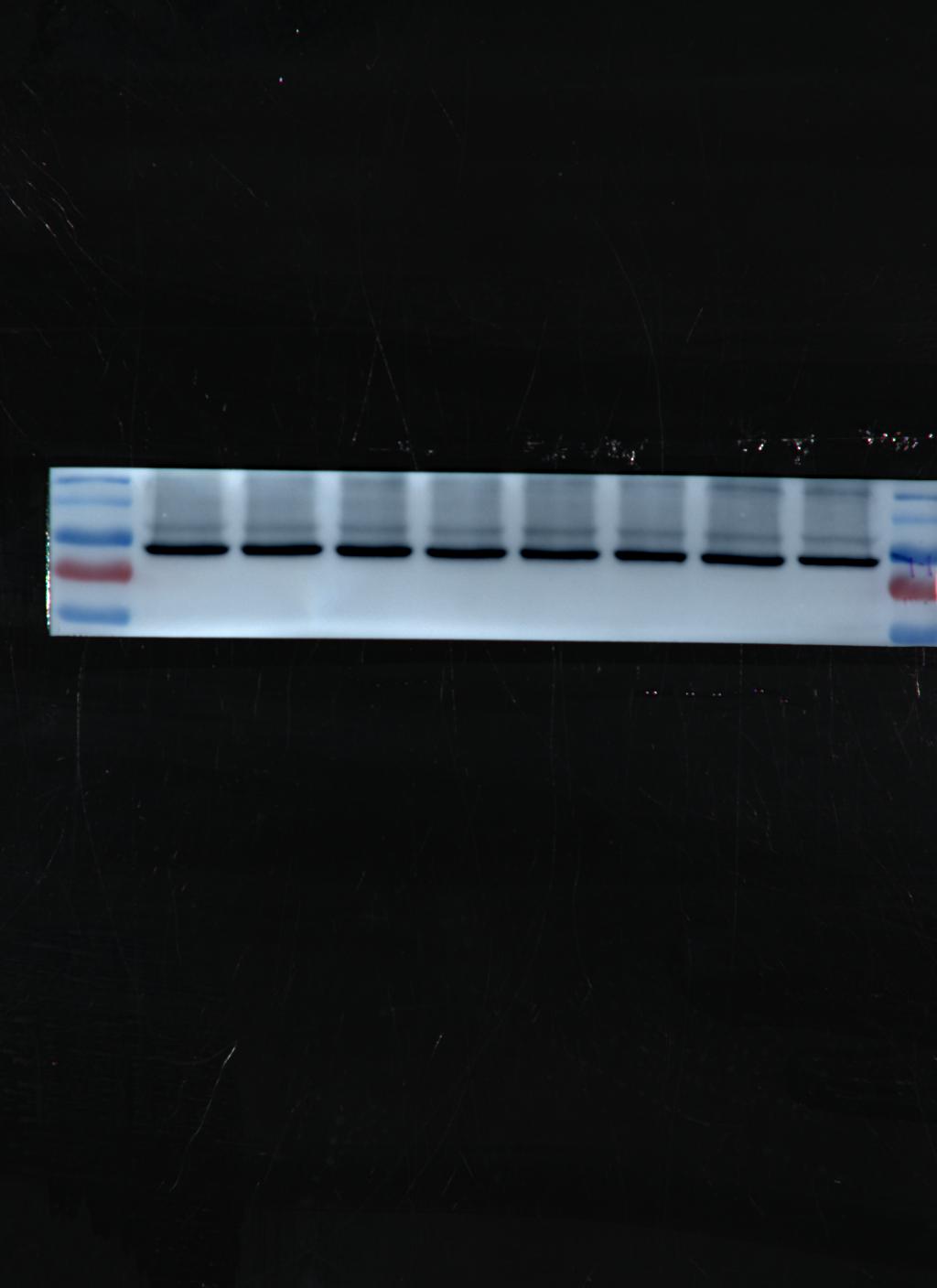

Supplement: Supplementary file 1 [file cancers-14-05188-s001.zip › WB(STING)/figure 2CTBK.jpg]

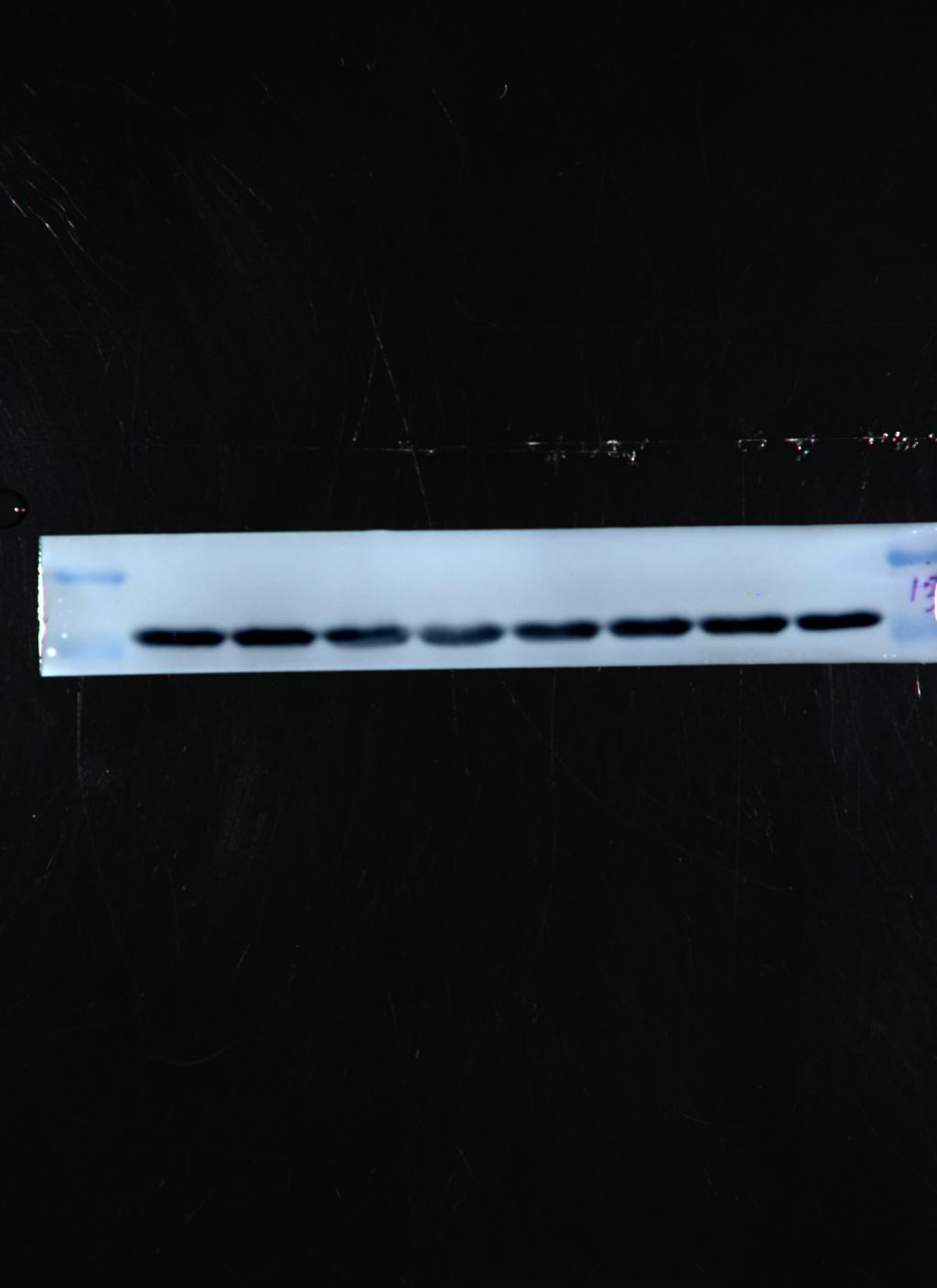

Supplement: Supplementary file 1 [file cancers-14-05188-s001.zip › WB(STING)/figure 2D gapdh.jpg]

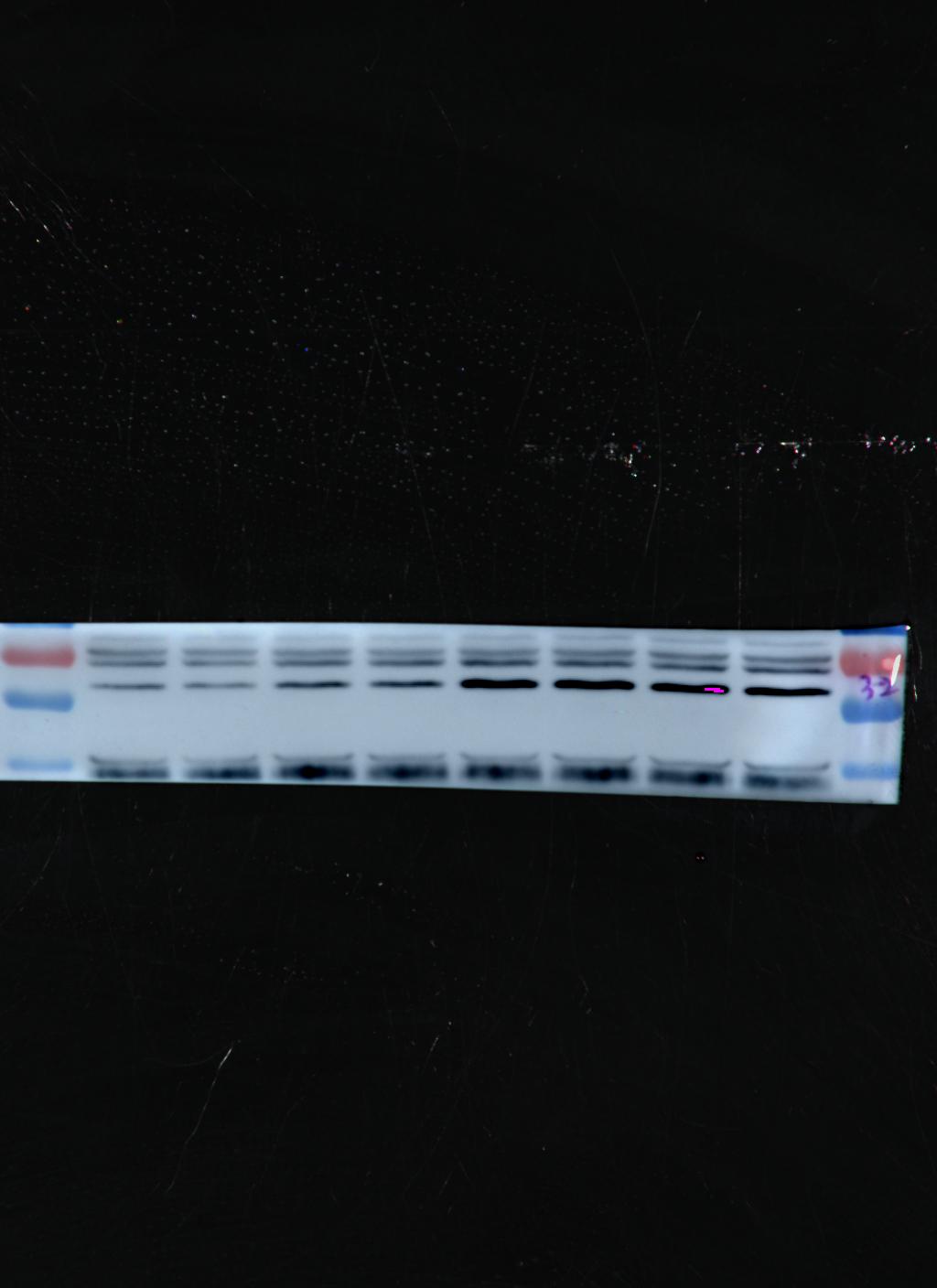

Supplement: Supplementary file 1 [file cancers-14-05188-s001.zip › WB(STING)/figure 2D nf-kb.jpg]

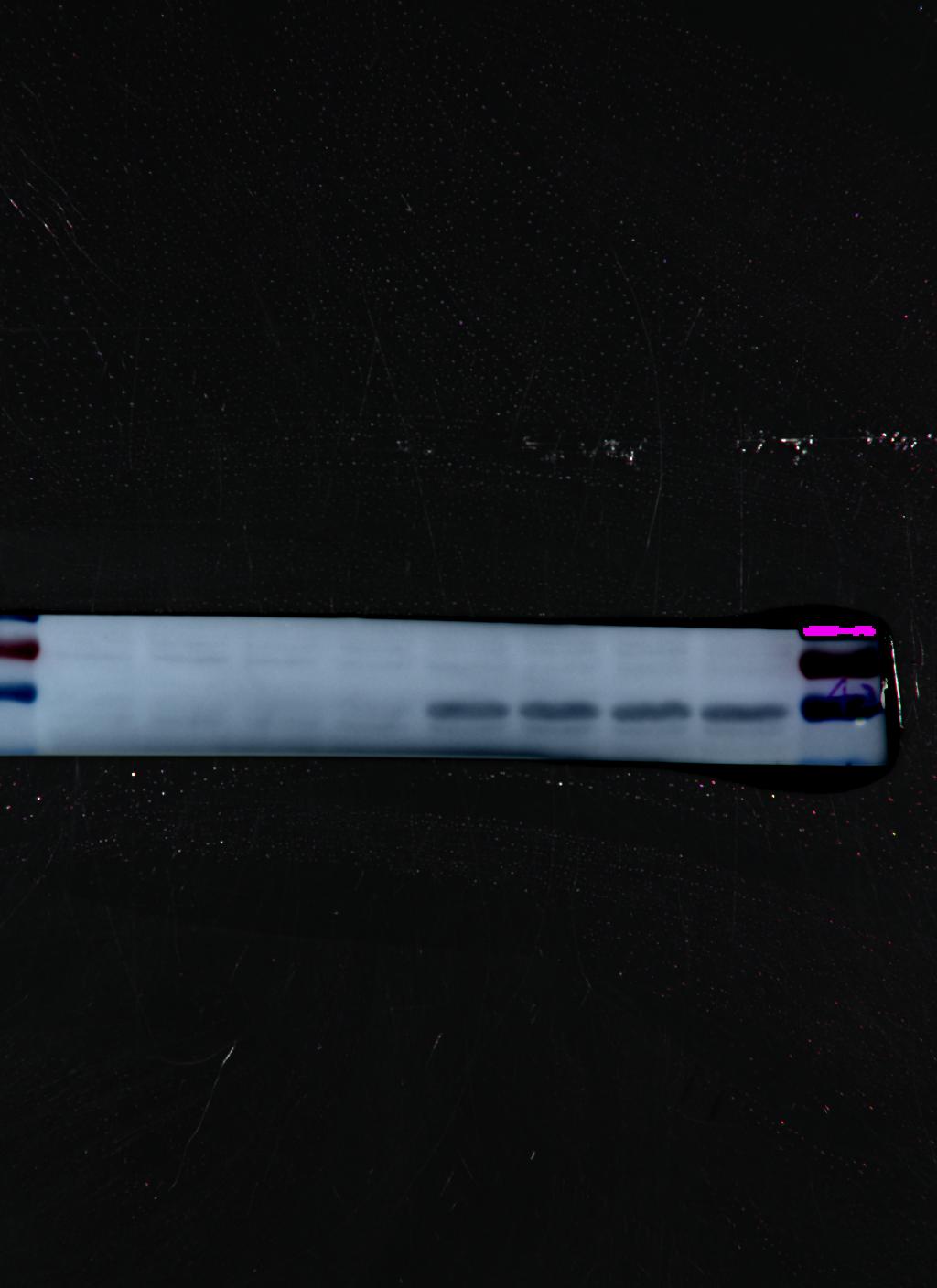

Supplement: Supplementary file 1 [file cancers-14-05188-s001.zip › WB(STING)/figure 2D p-NF-kB.jpg]

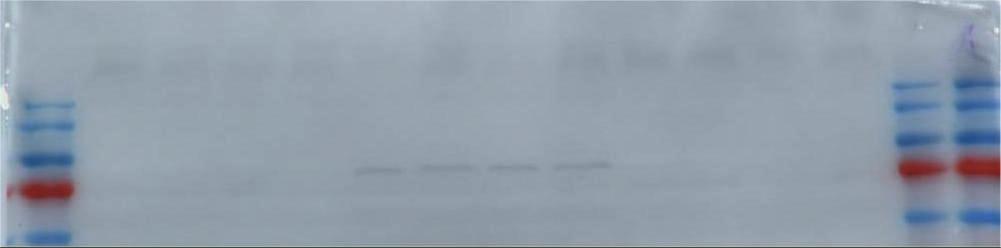

Supplement: Supplementary file 1 [file cancers-14-05188-s001.zip › WB(STING)/figure 3Cp-TBK1.jpg]

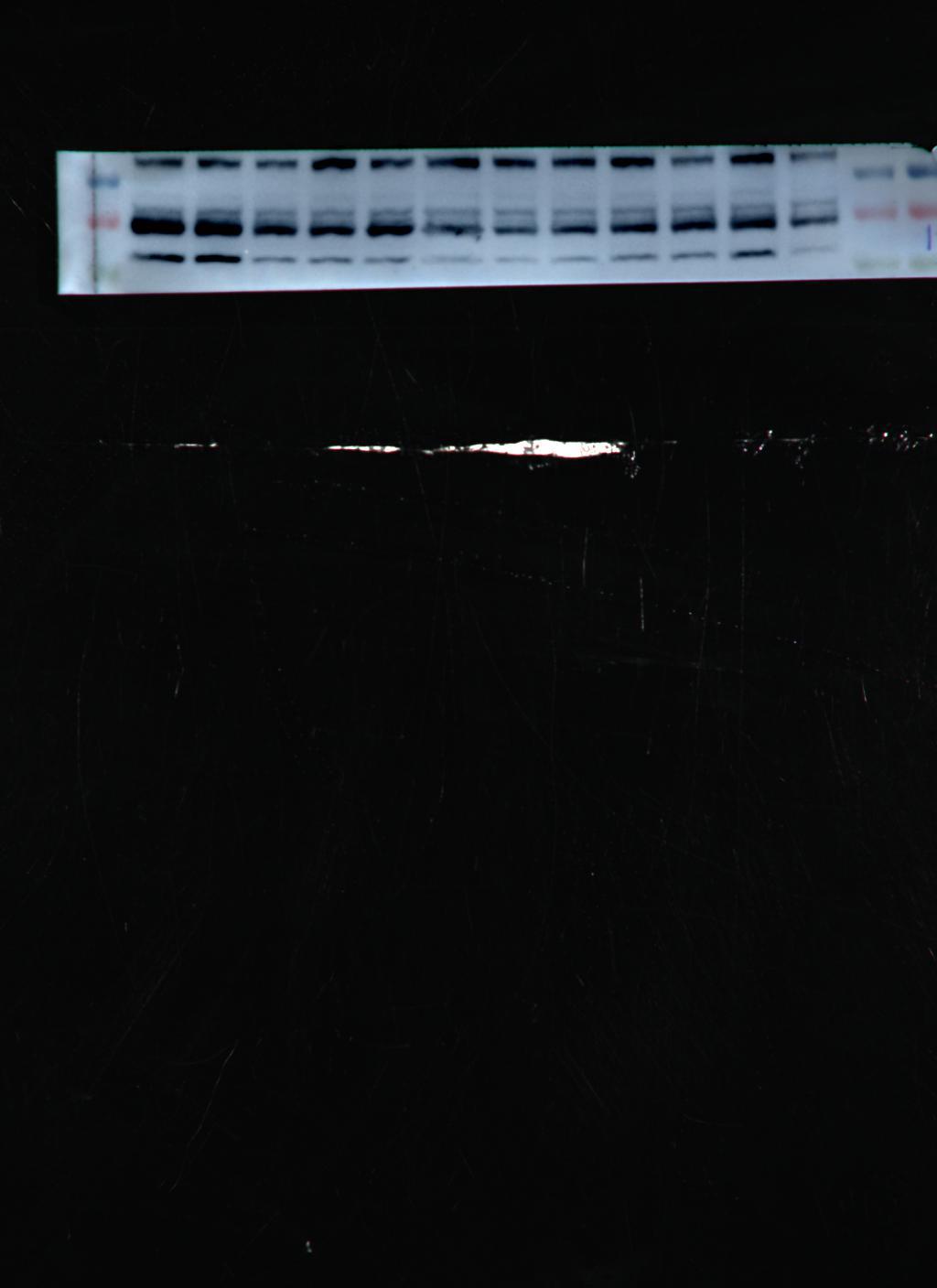

Supplement: Supplementary file 1 [file cancers-14-05188-s001.zip › WB(STING)/figure 3D NF-kB.jpg]

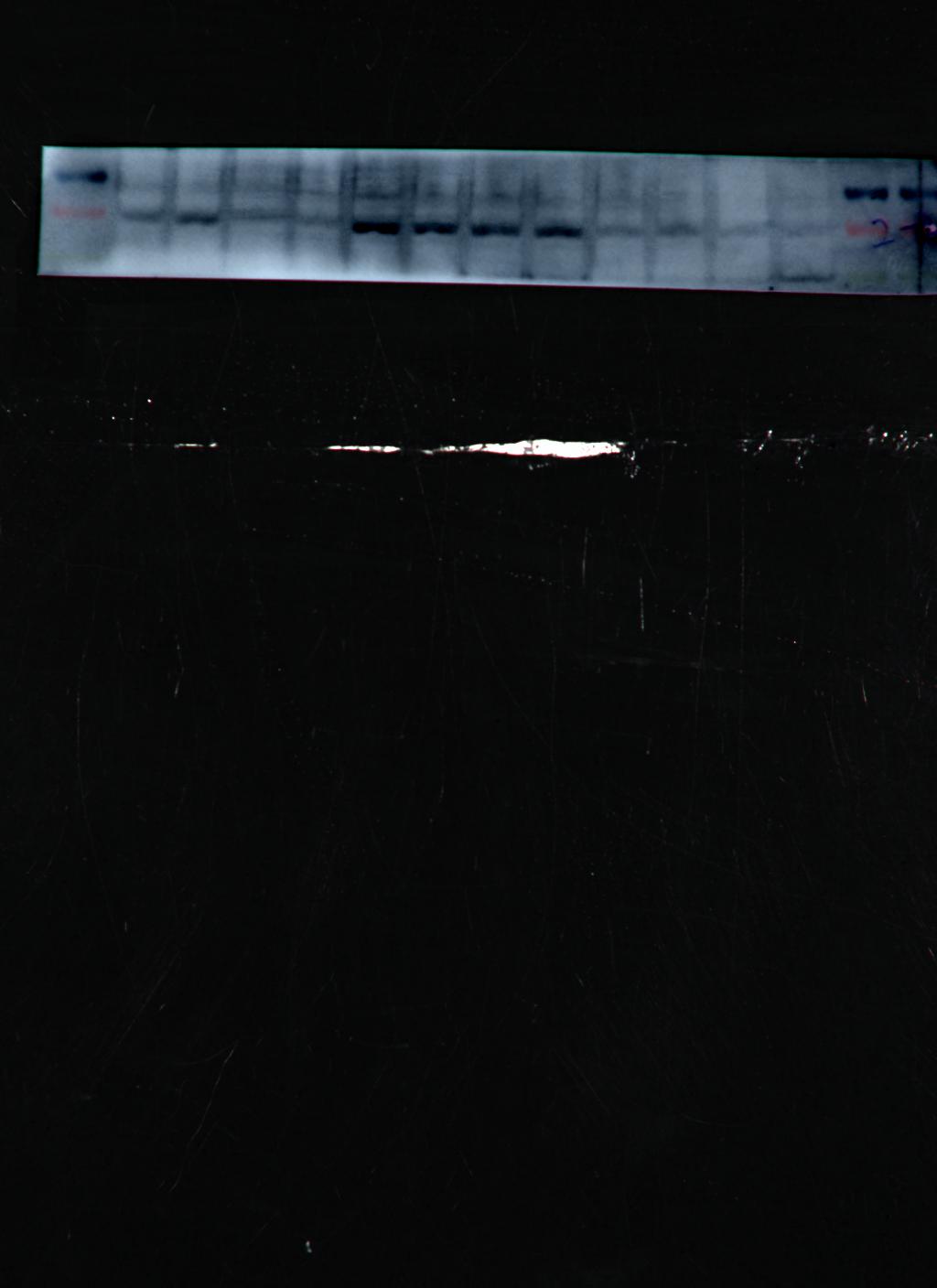

Supplement: Supplementary file 1 [file cancers-14-05188-s001.zip › WB(STING)/figure 3D p-NF-kB.jpg]

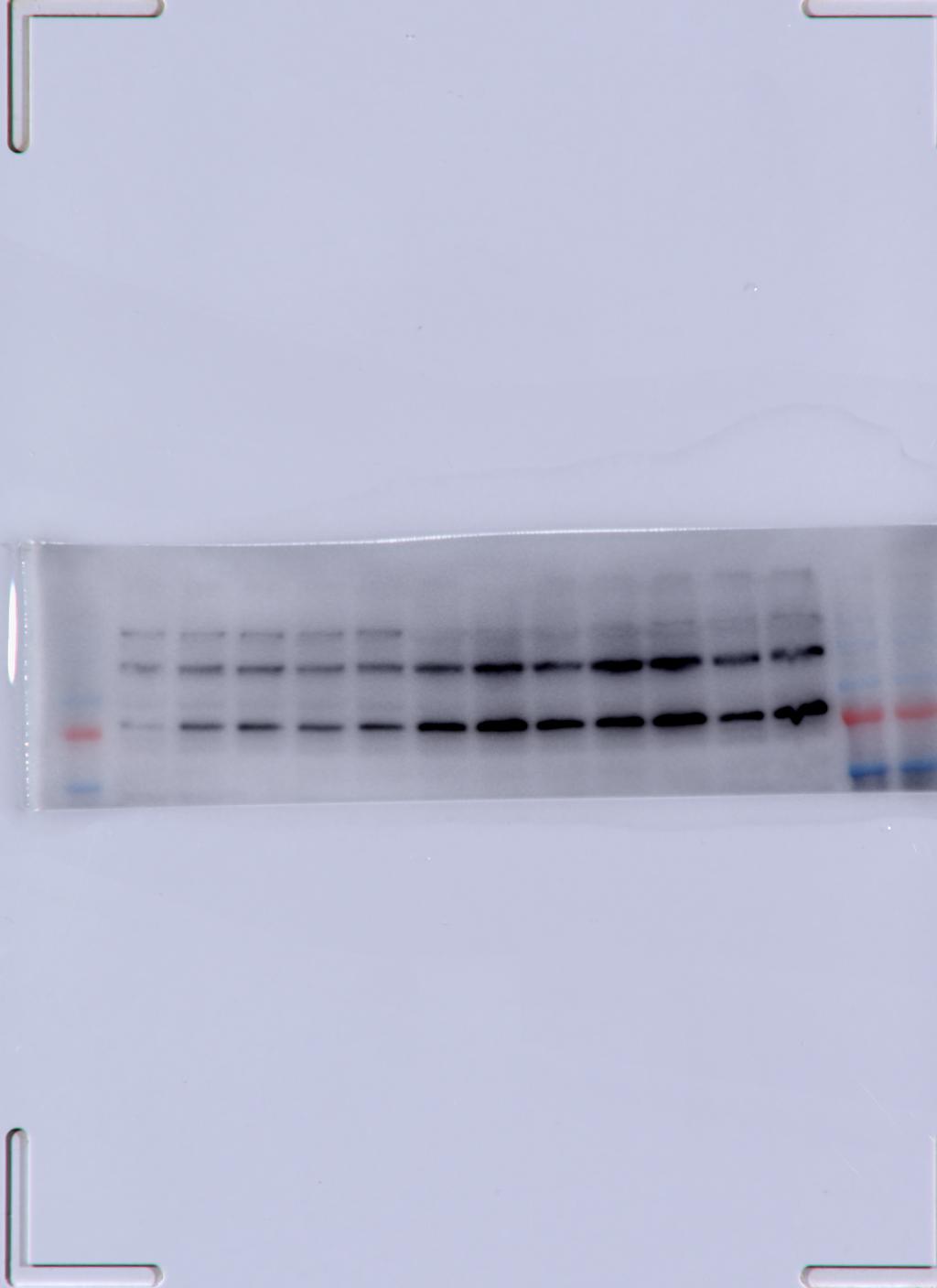

Supplement: Supplementary file 1 [file cancers-14-05188-s001.zip › WB(STING)/figure 4 D CD206.jpg]

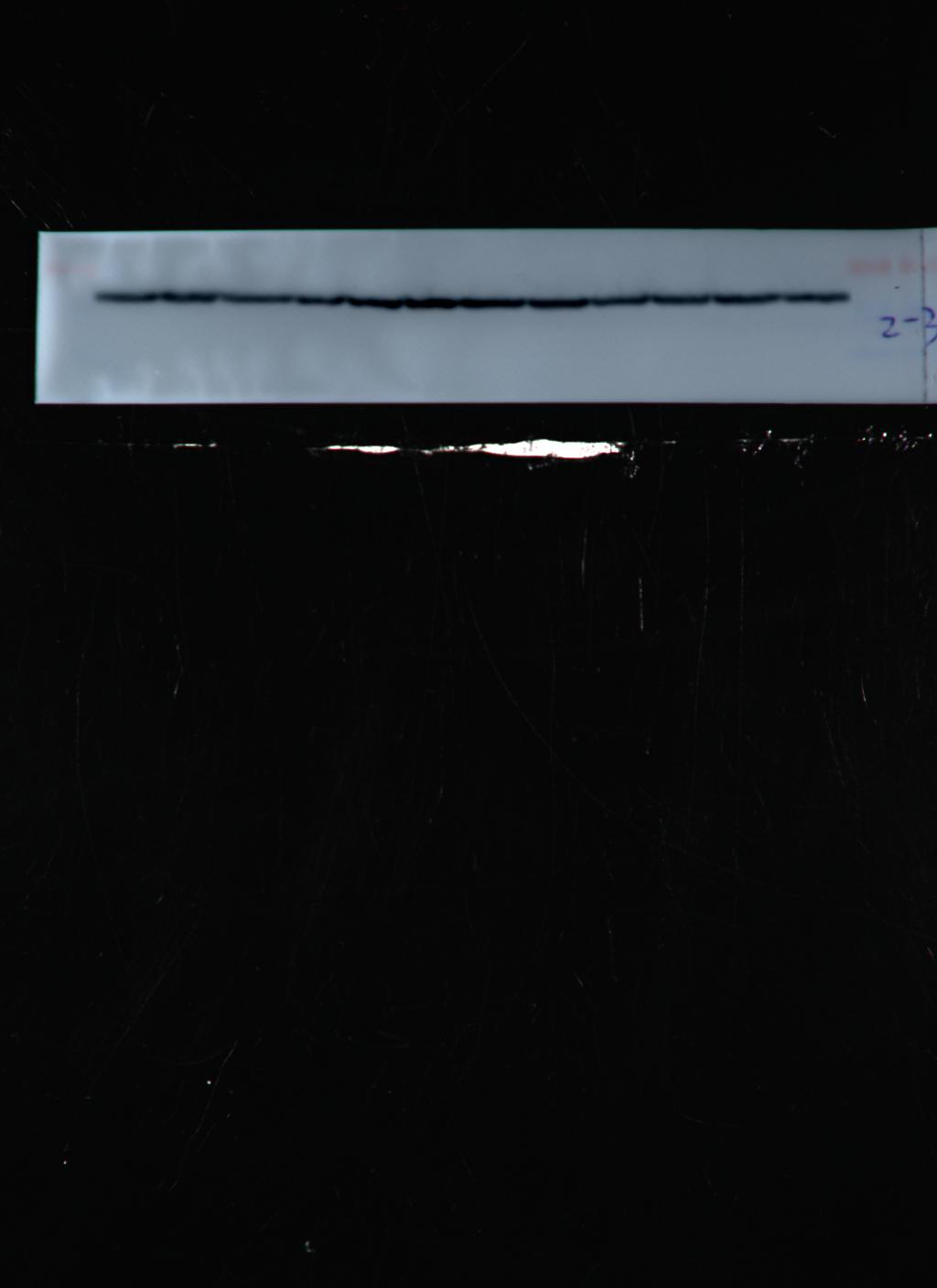

Supplement: Supplementary file 1 [file cancers-14-05188-s001.zip › WB(STING)/figure 4D GAPDH.jpg]

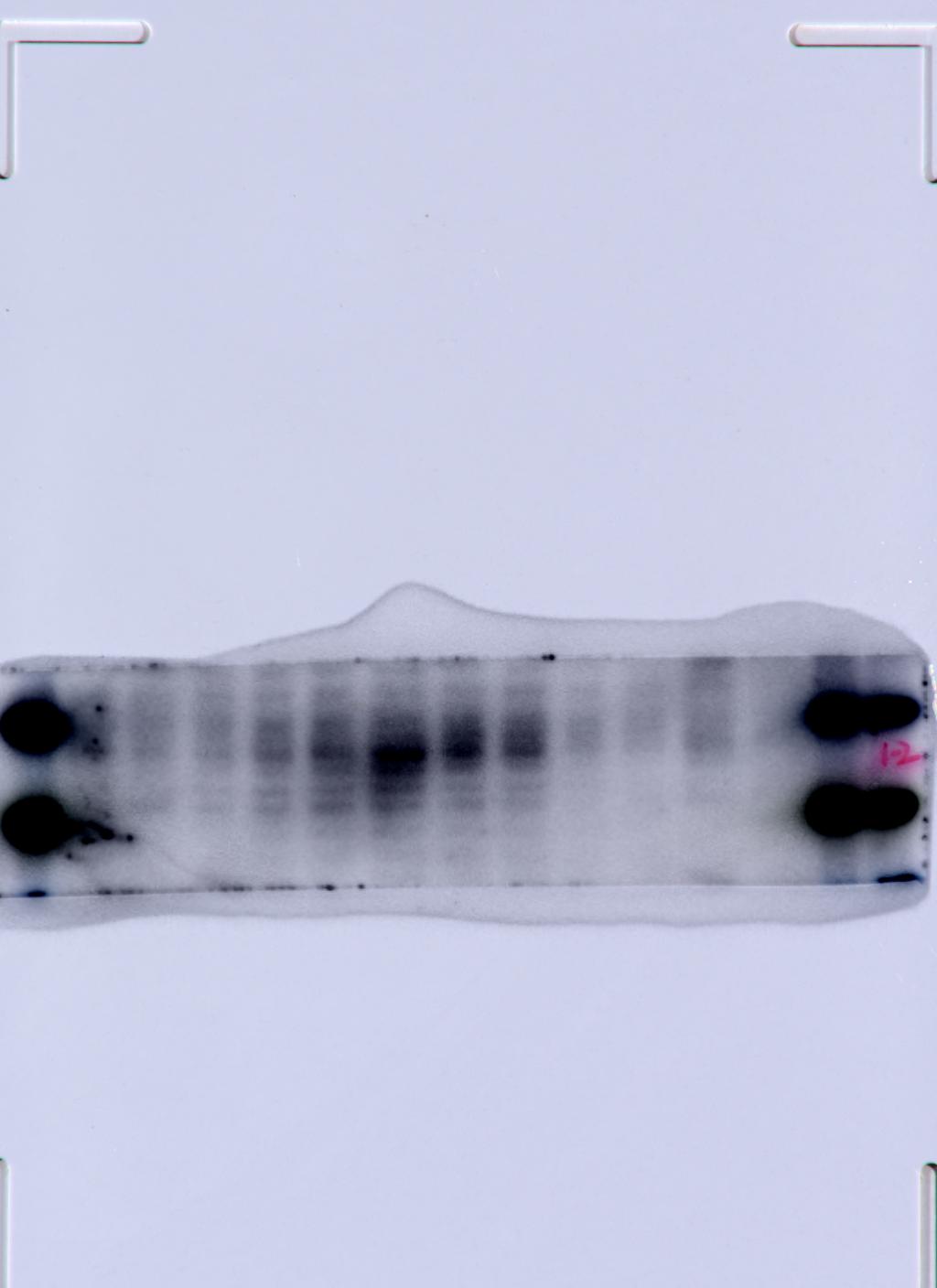

Supplement: Supplementary file 1 [file cancers-14-05188-s001.zip › WB(STING)/Figure 4E IL-1ß║₧.jpg]

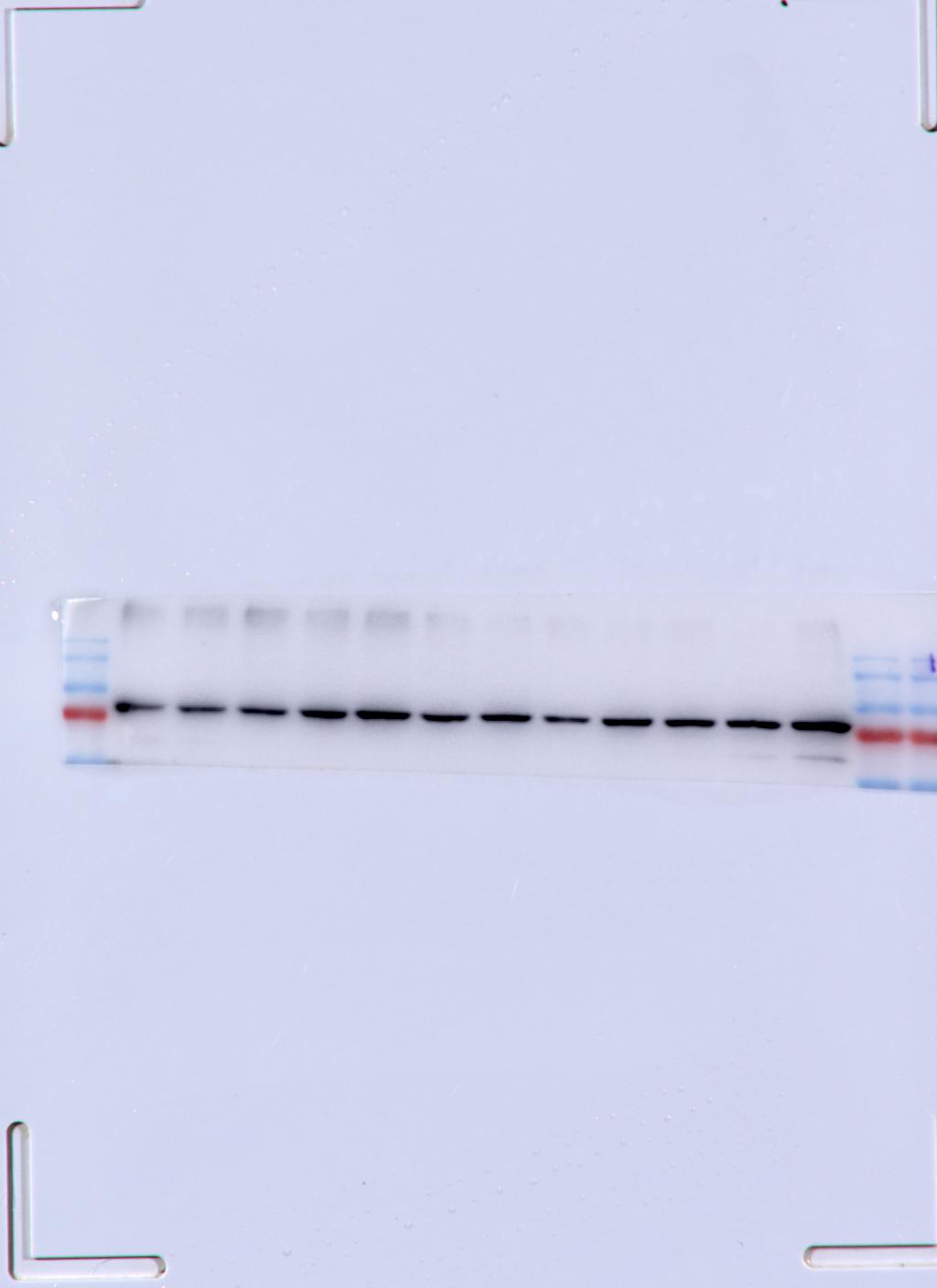

Supplement: Supplementary file 1 [file cancers-14-05188-s001.zip › WB(STING)/figure 5 D TBK1.jpg]

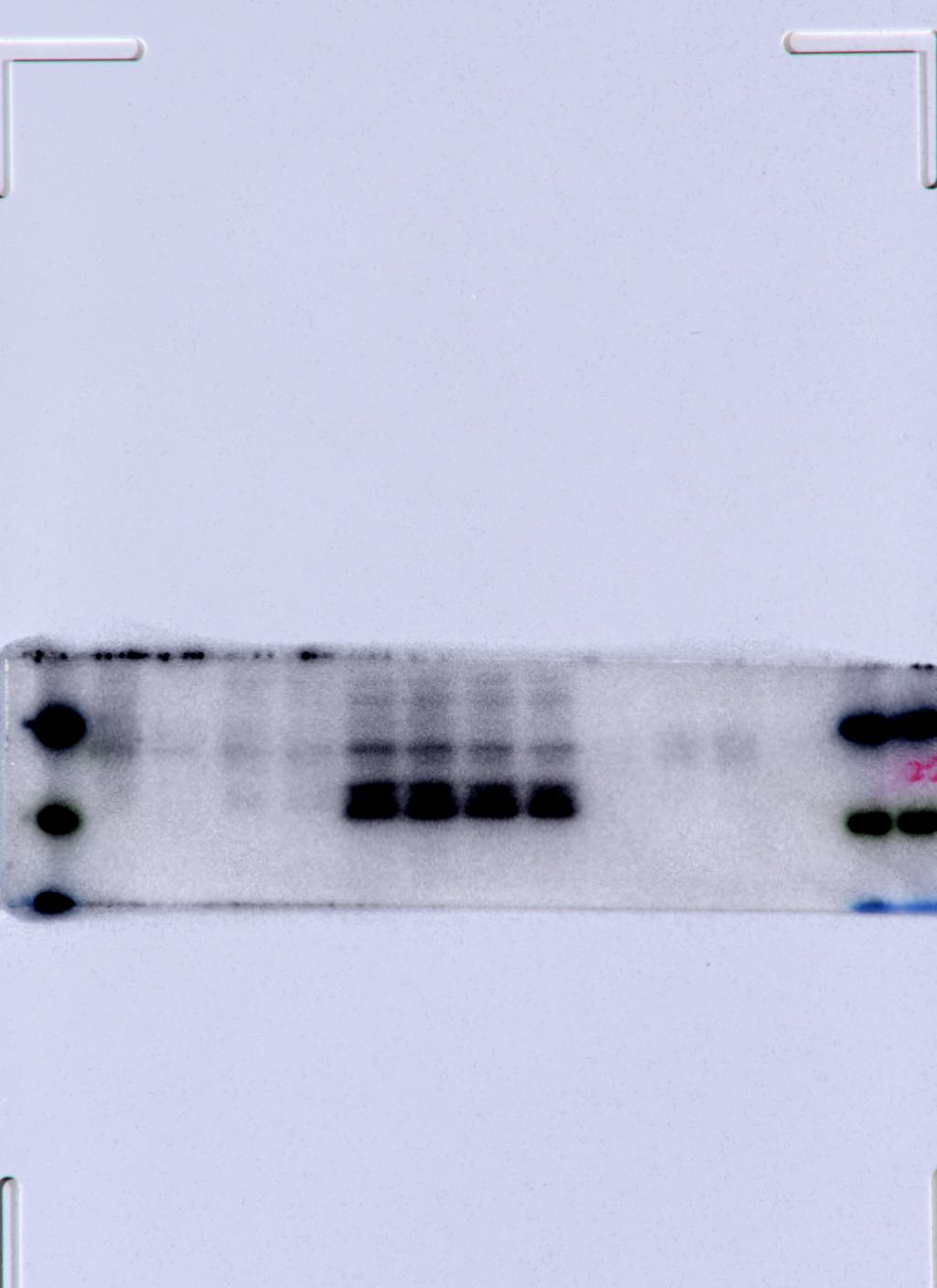

Supplement: Supplementary file 1 [file cancers-14-05188-s001.zip › WB(STING)/Figure 5 E IL-ß║₧.jpg]

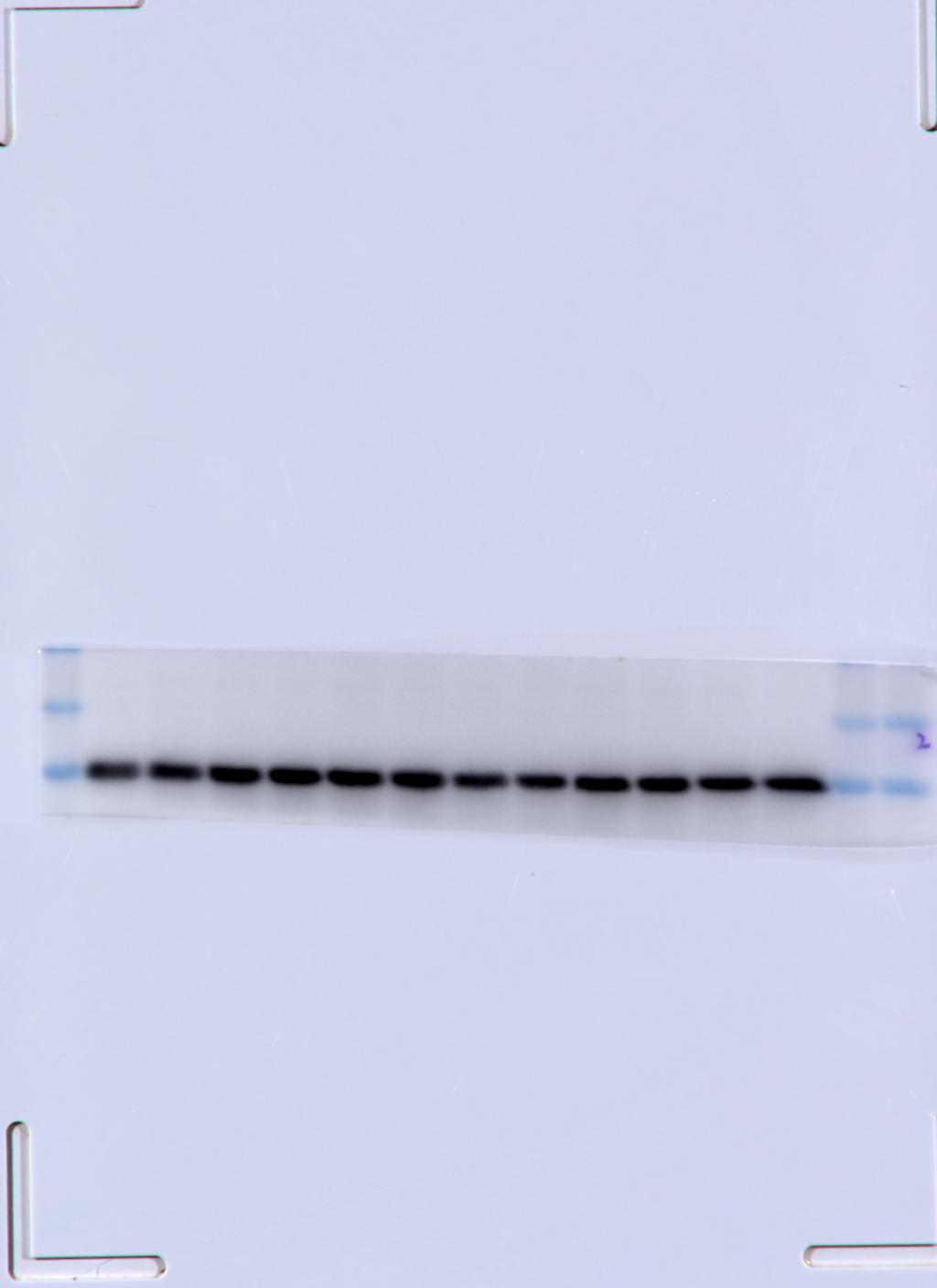

Supplement: Supplementary file 1 [file cancers-14-05188-s001.zip › WB(STING)/figure 5B GAPDH.jpg]

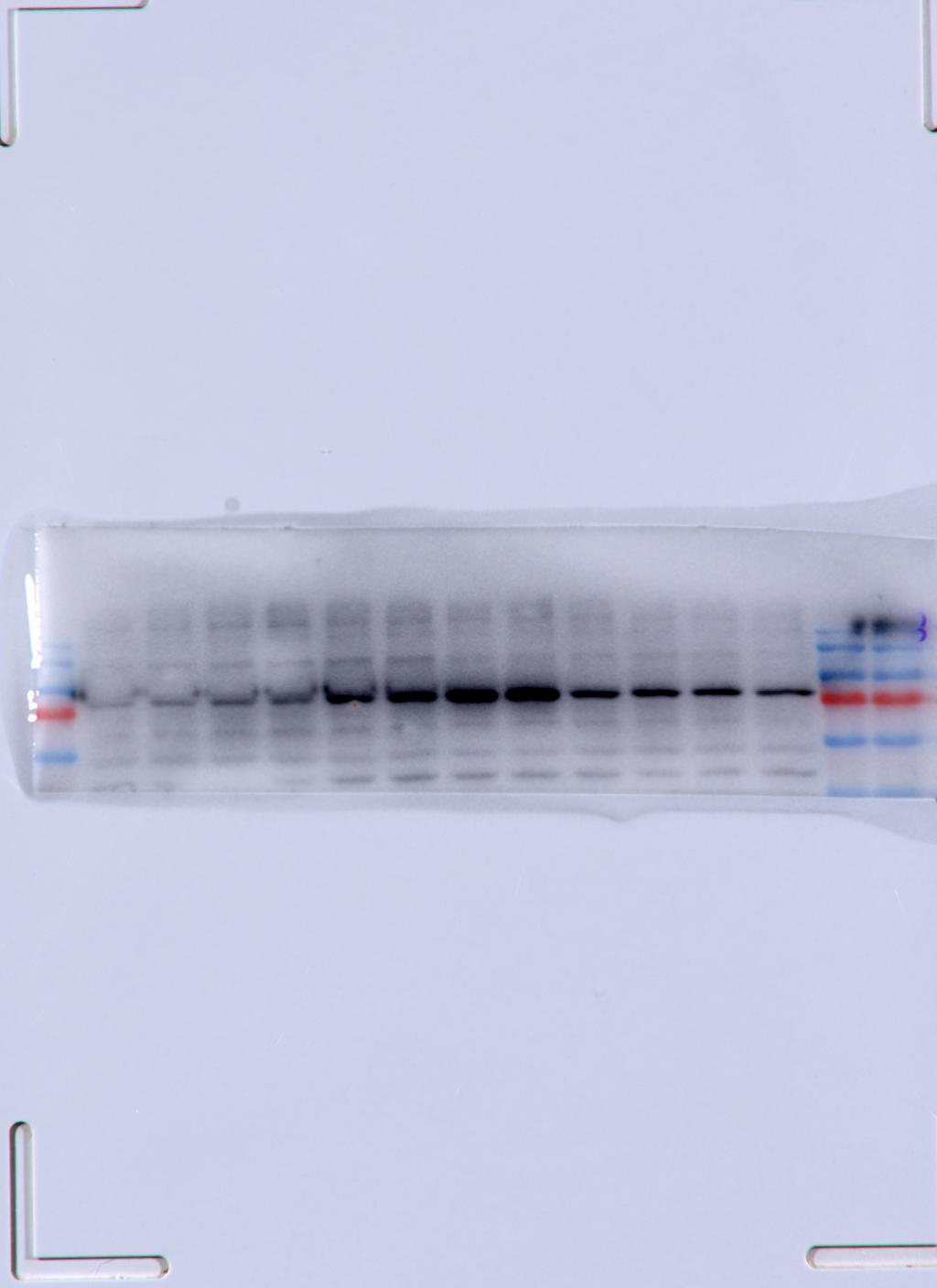

Supplement: Supplementary file 1 [file cancers-14-05188-s001.zip › WB(STING)/figure 5C p-tbk1.jpg]

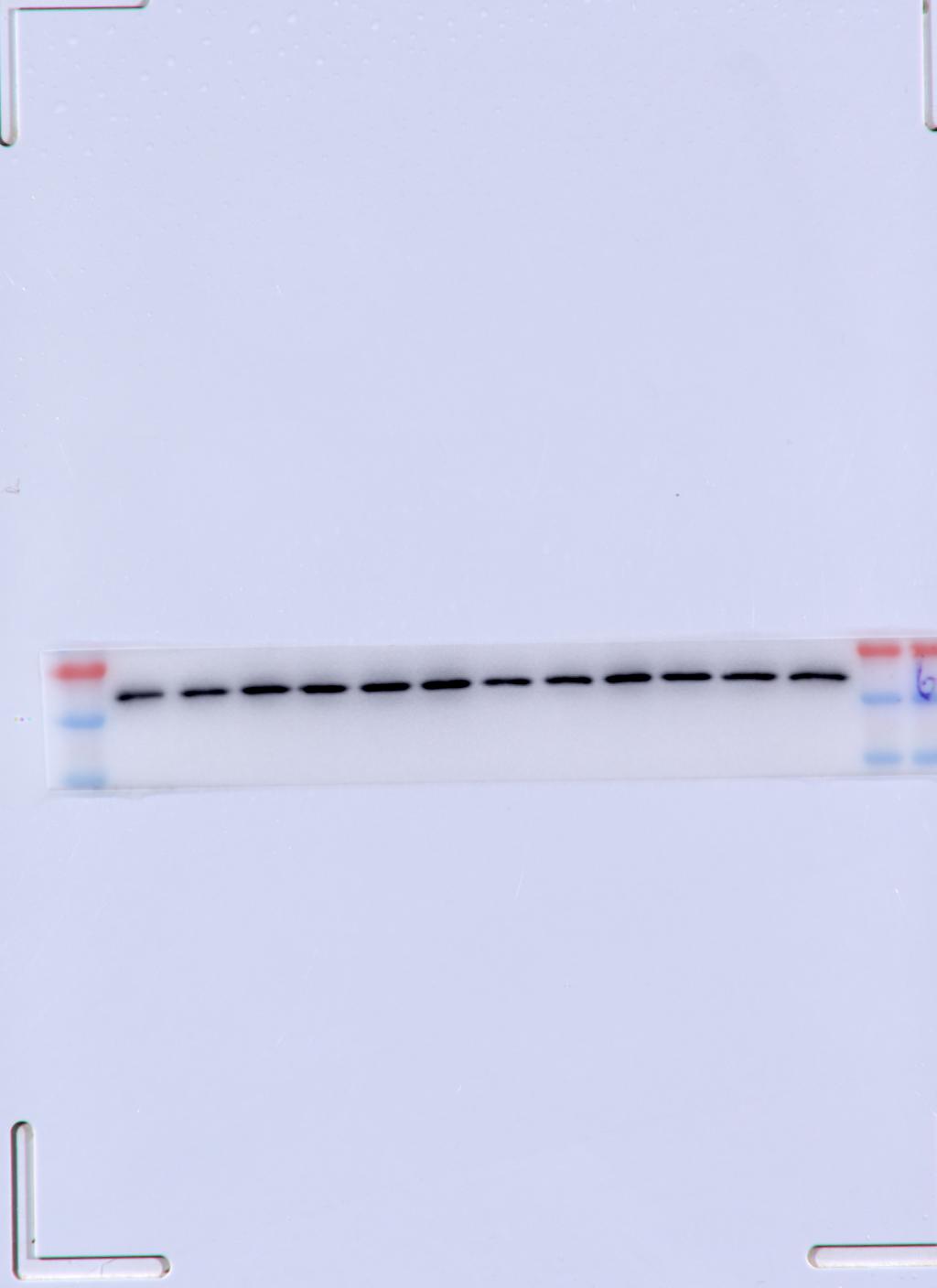

Supplement: Supplementary file 1 [file cancers-14-05188-s001.zip › WB(STING)/figure 5C-nf-kb-1.jpg]

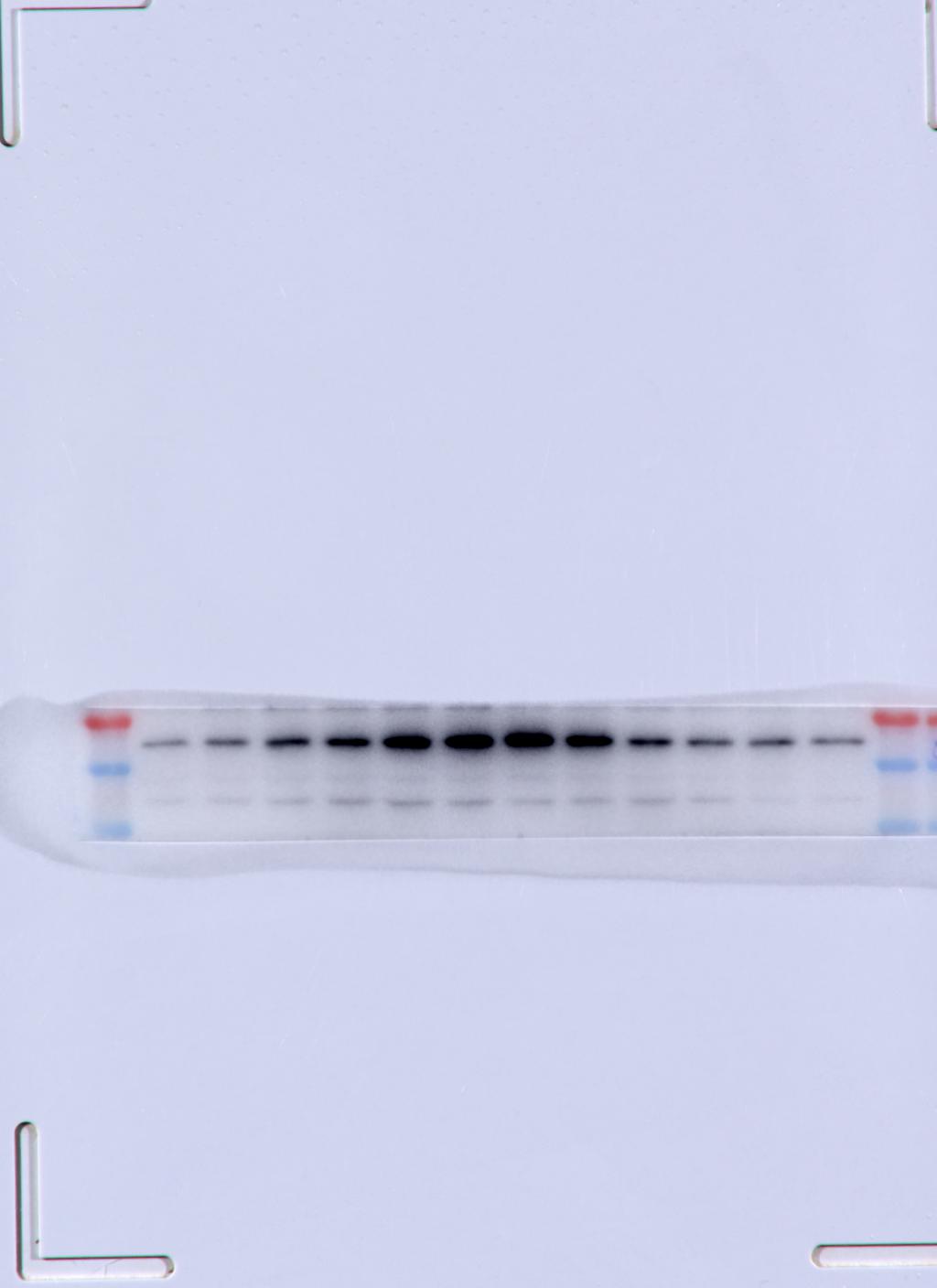

Supplement: Supplementary file 1 [file cancers-14-05188-s001.zip › WB(STING)/figure 5Cp-nf-kb.jpg]

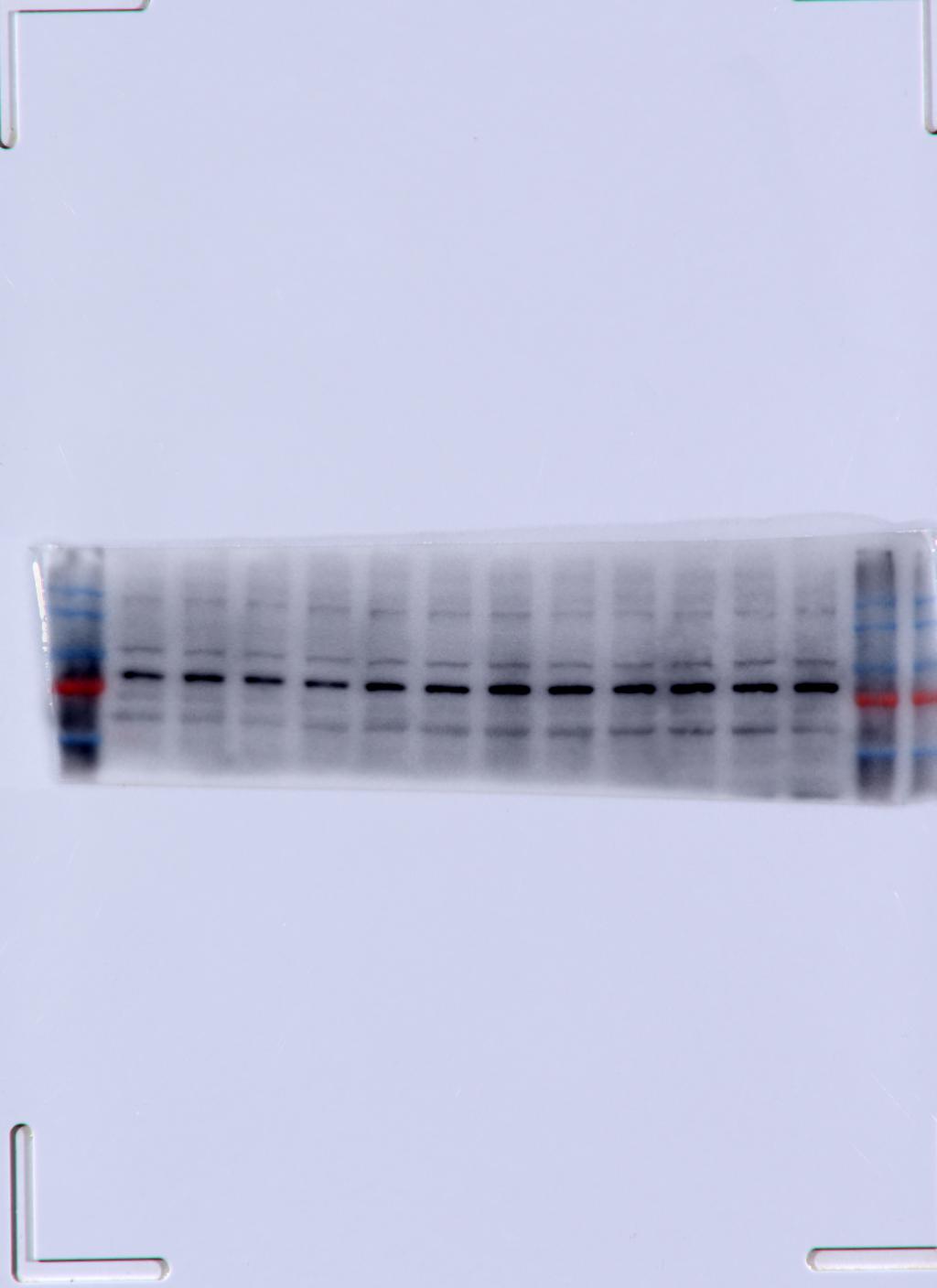

Supplement: Supplementary file 1 [file cancers-14-05188-s001.zip › WB(STING)/figure 5D CD206.jpg]

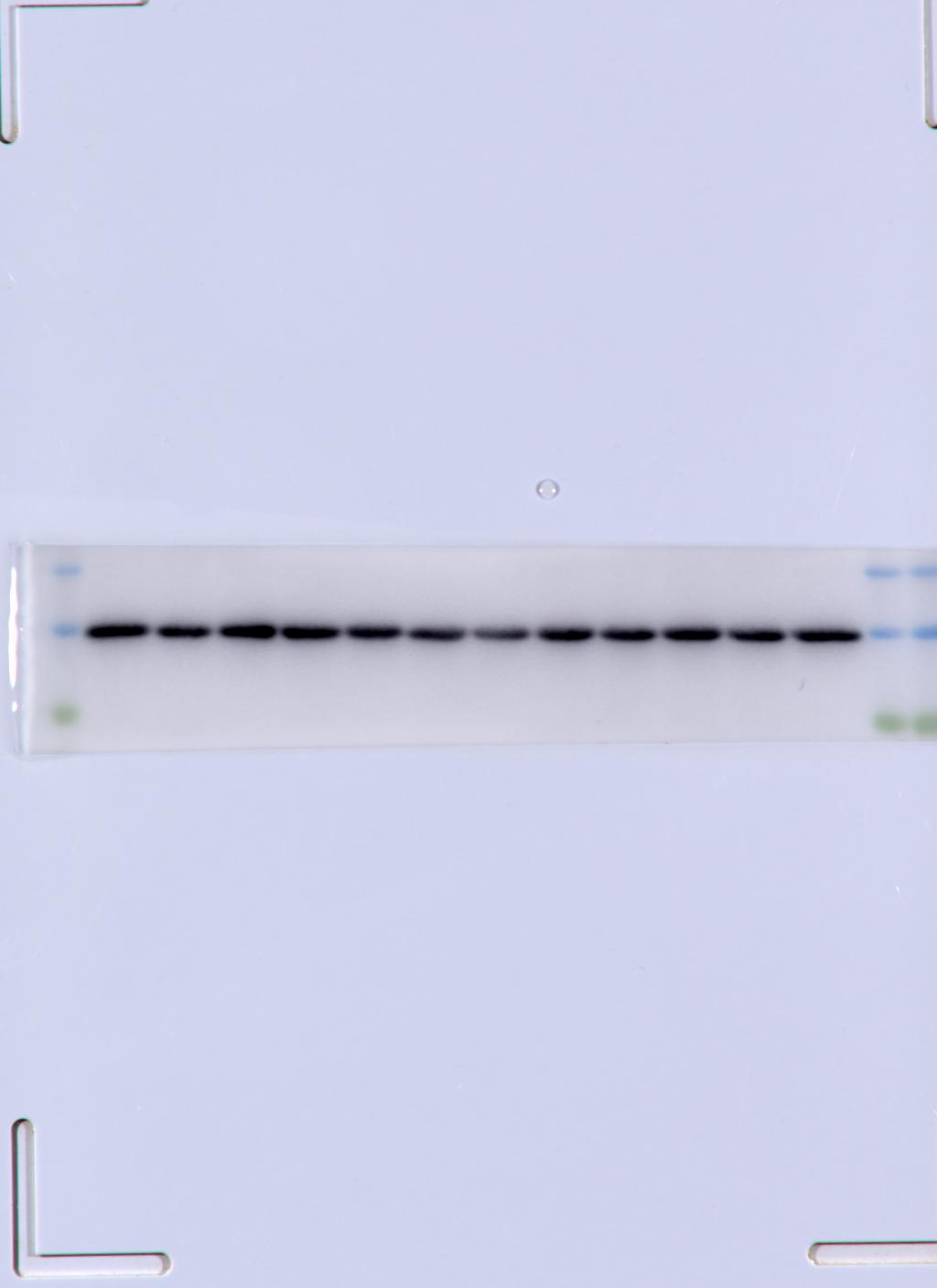

Supplement: Supplementary file 1 [file cancers-14-05188-s001.zip › WB(STING)/figure 5D GAPDH.jpg]

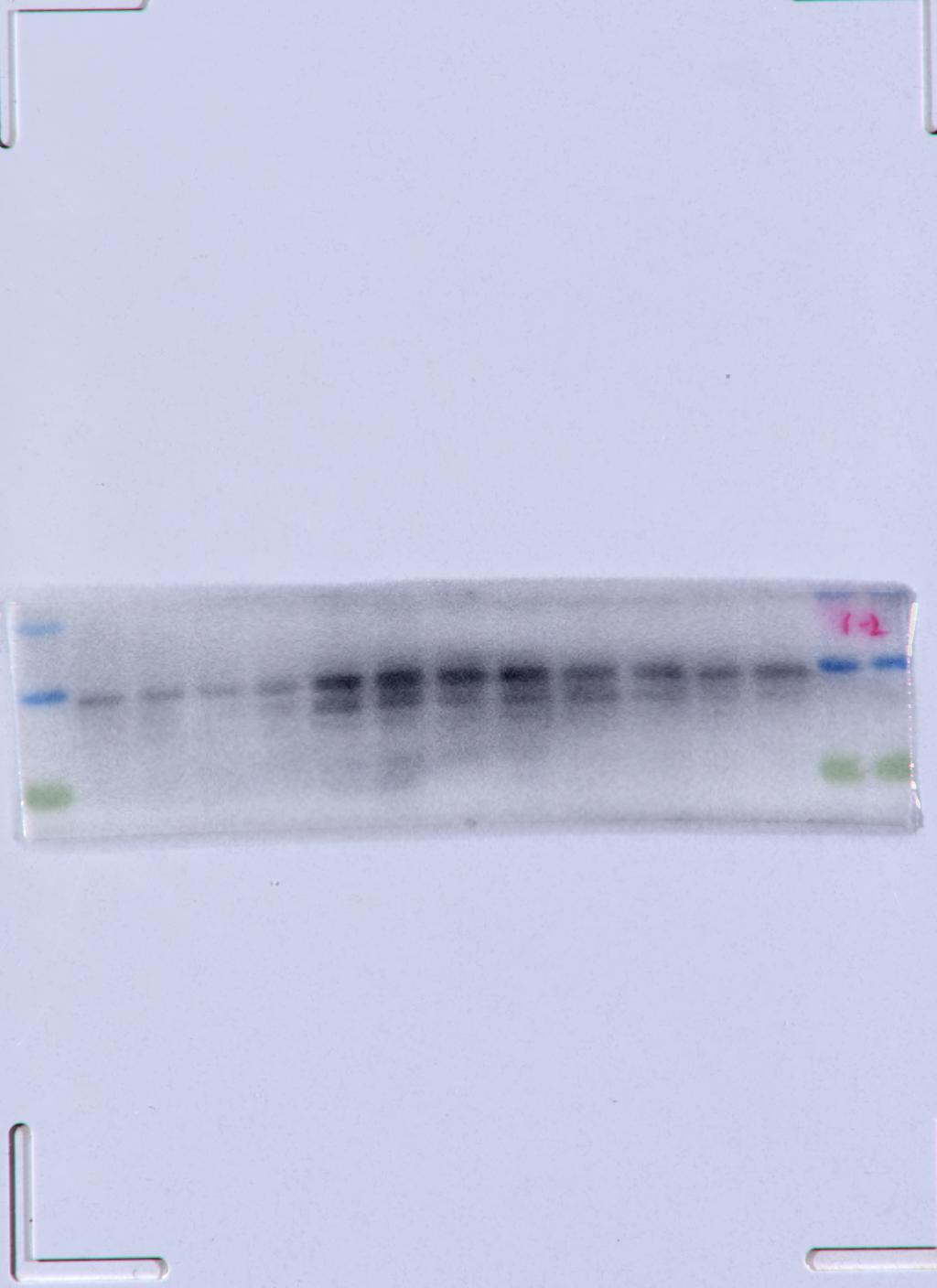

Supplement: Supplementary file 1 [file cancers-14-05188-s001.zip › WB(STING)/figure 5D MHCII.jpg]

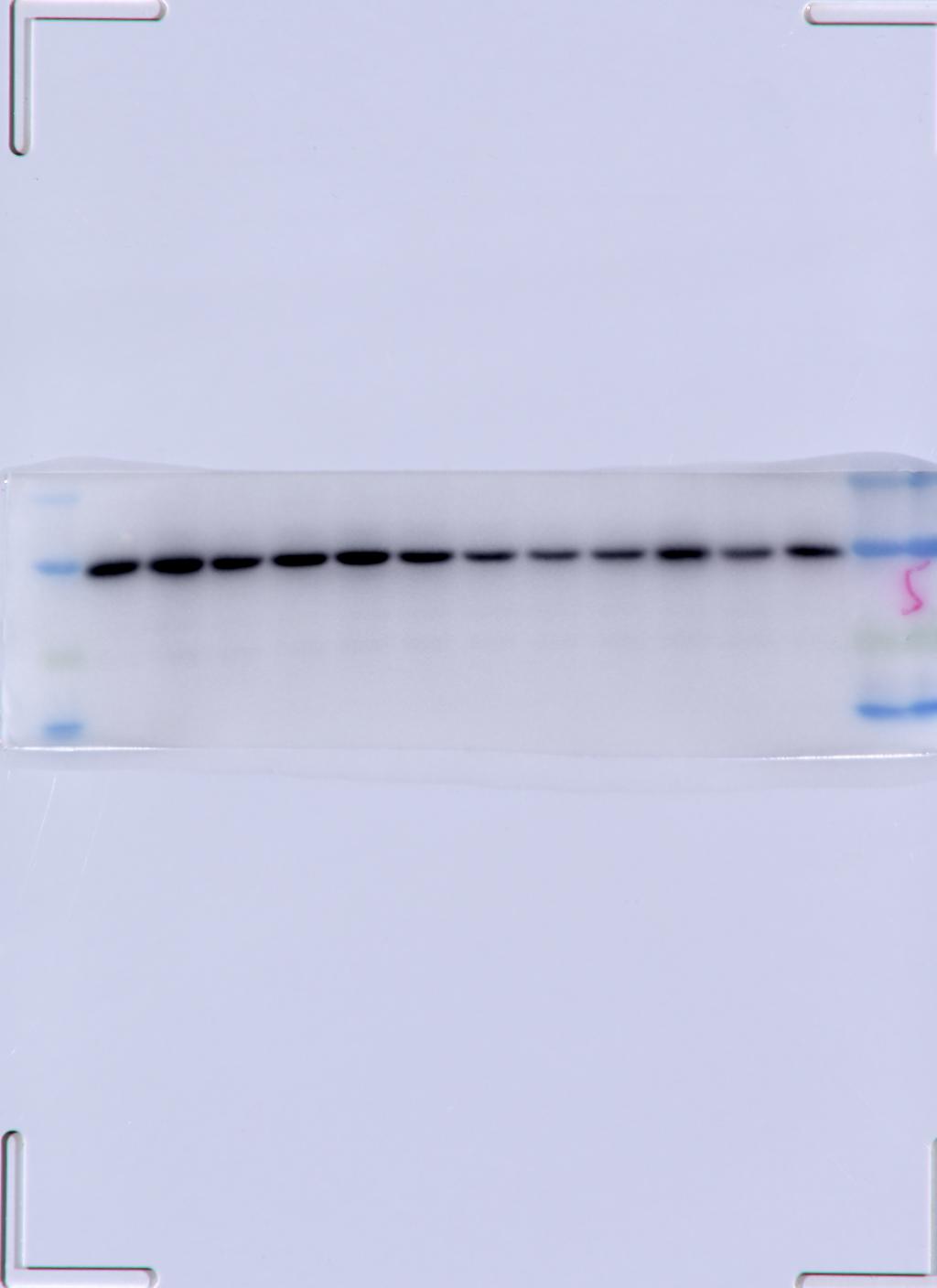

Supplement: Supplementary file 1 [file cancers-14-05188-s001.zip › WB(STING)/Figure 5EGAPDH.jpg]

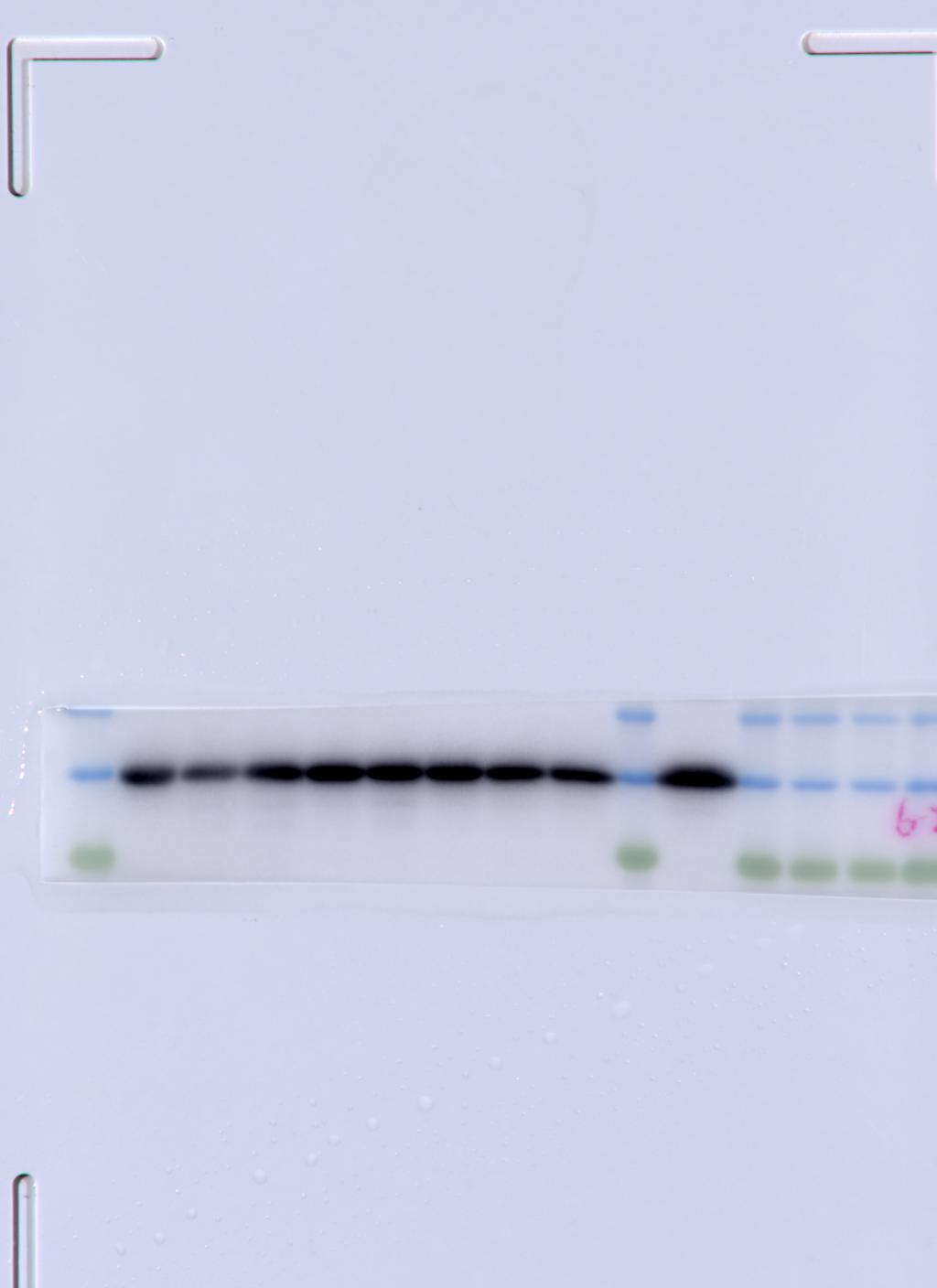

Supplement: Supplementary file 1 [file cancers-14-05188-s001.zip › WB(STING)/fIGURE1 E GAPDH.jpg]

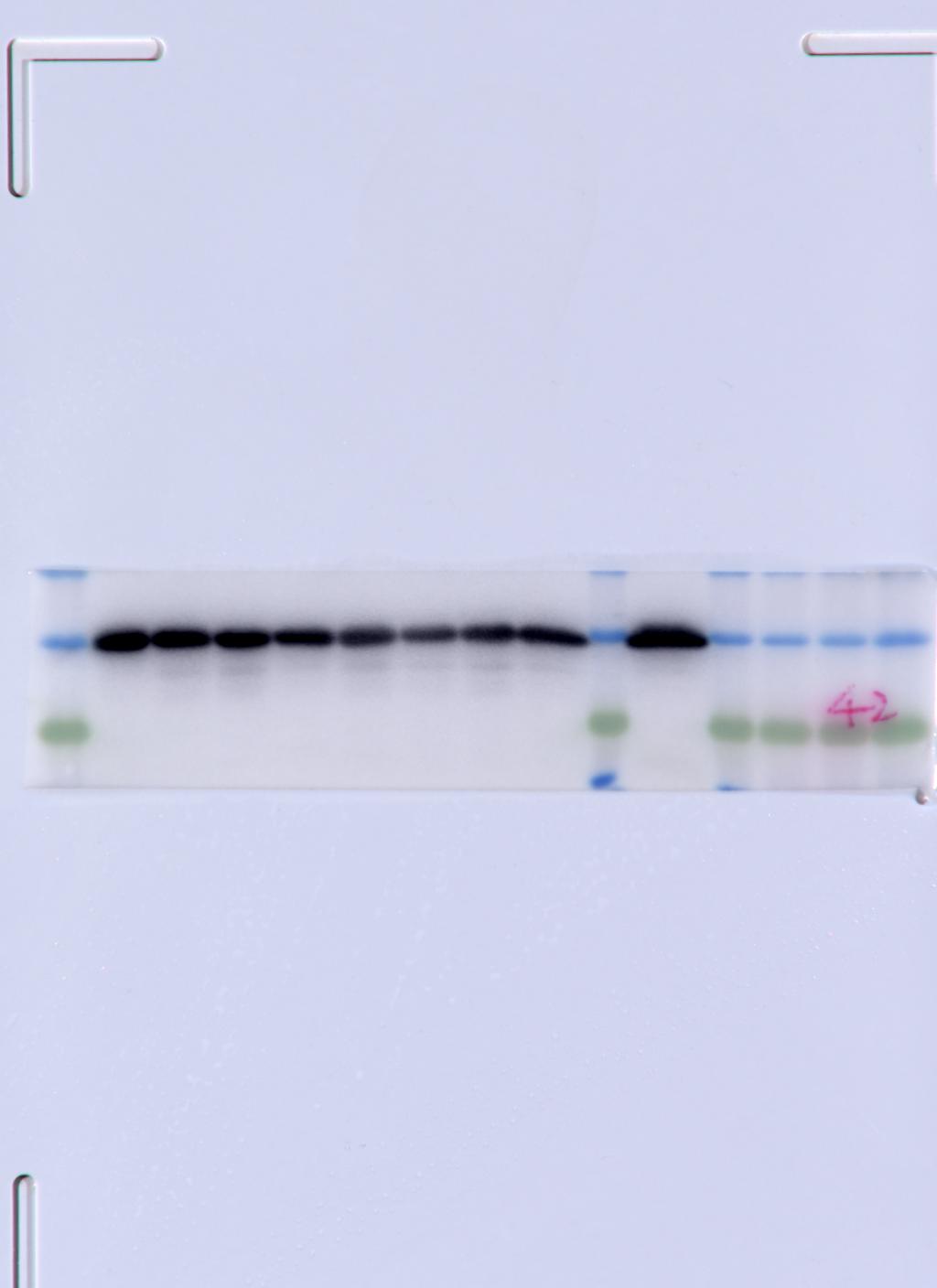

Supplement: Supplementary file 1 [file cancers-14-05188-s001.zip › WB(STING)/Figure1 EGAPDH.jpg]

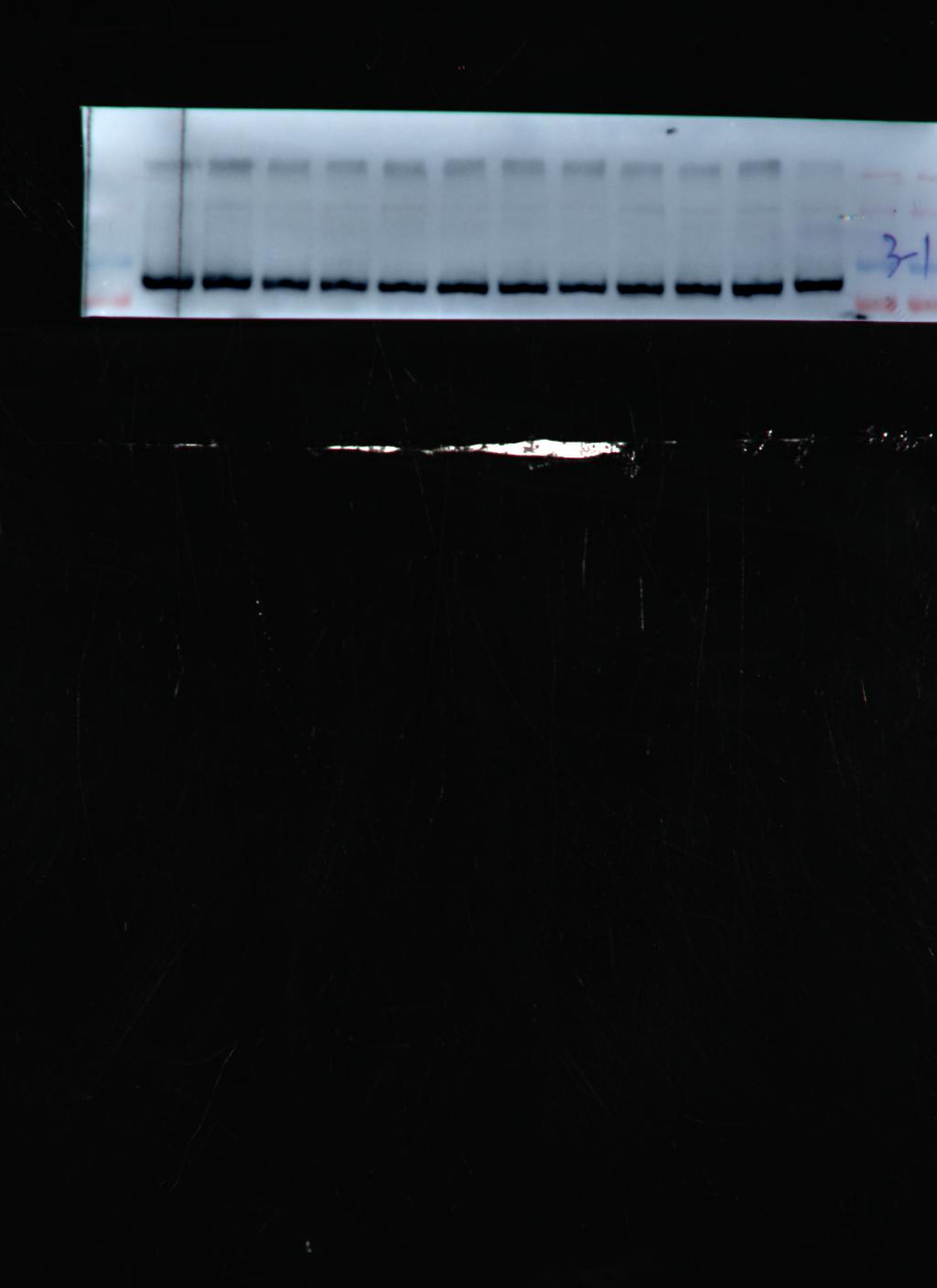

Supplement: Supplementary file 1 [file cancers-14-05188-s001.zip › WB(STING)/figure3C TBK1.jpg]

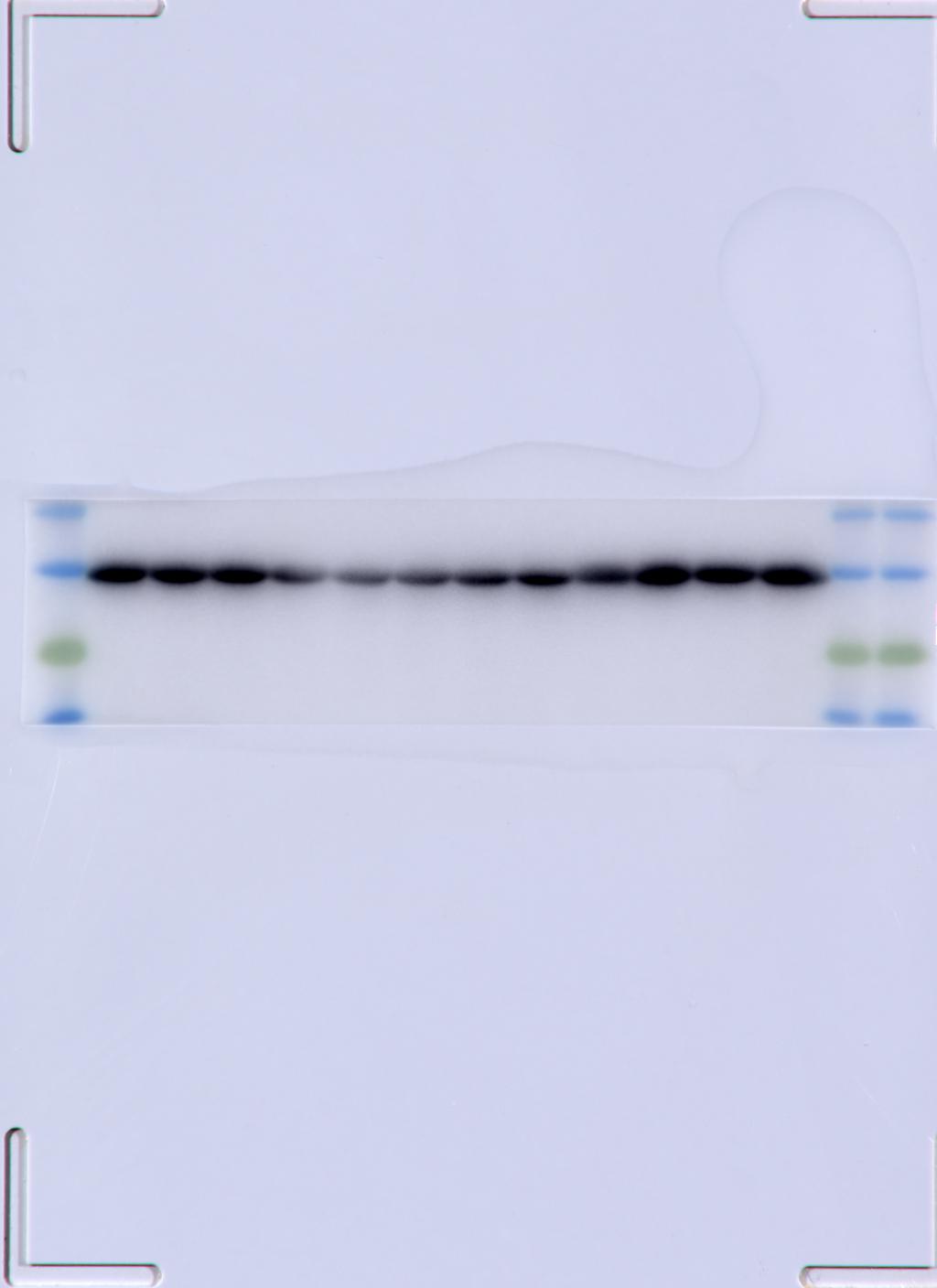

Supplement: Supplementary file 1 [file cancers-14-05188-s001.zip › WB(STING)/Figure4 EGAPDH.jpg]

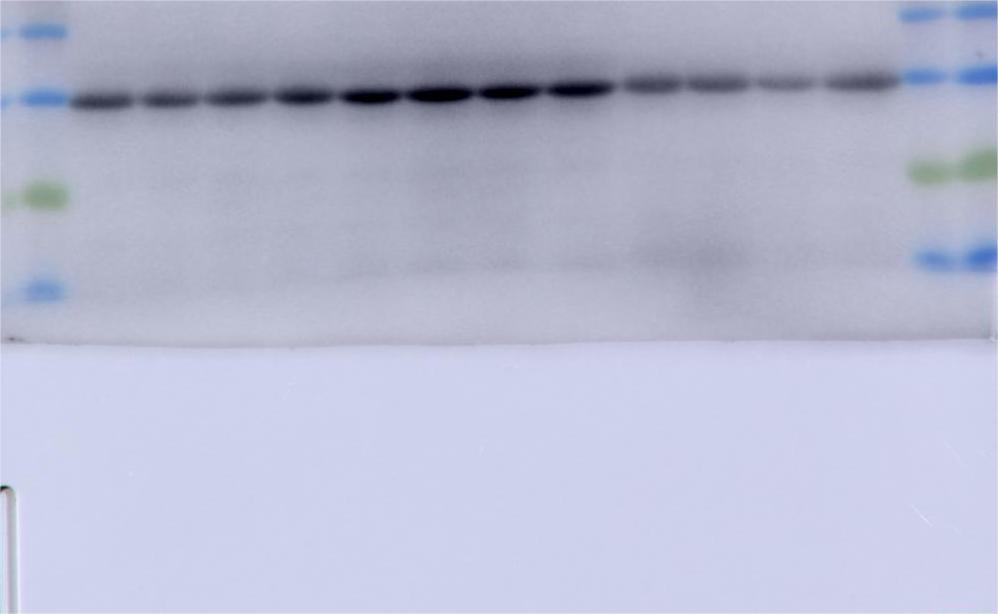

Supplement: Supplementary file 1 [file cancers-14-05188-s001.zip › WB(STING)/figure4D MHCII.jpg]

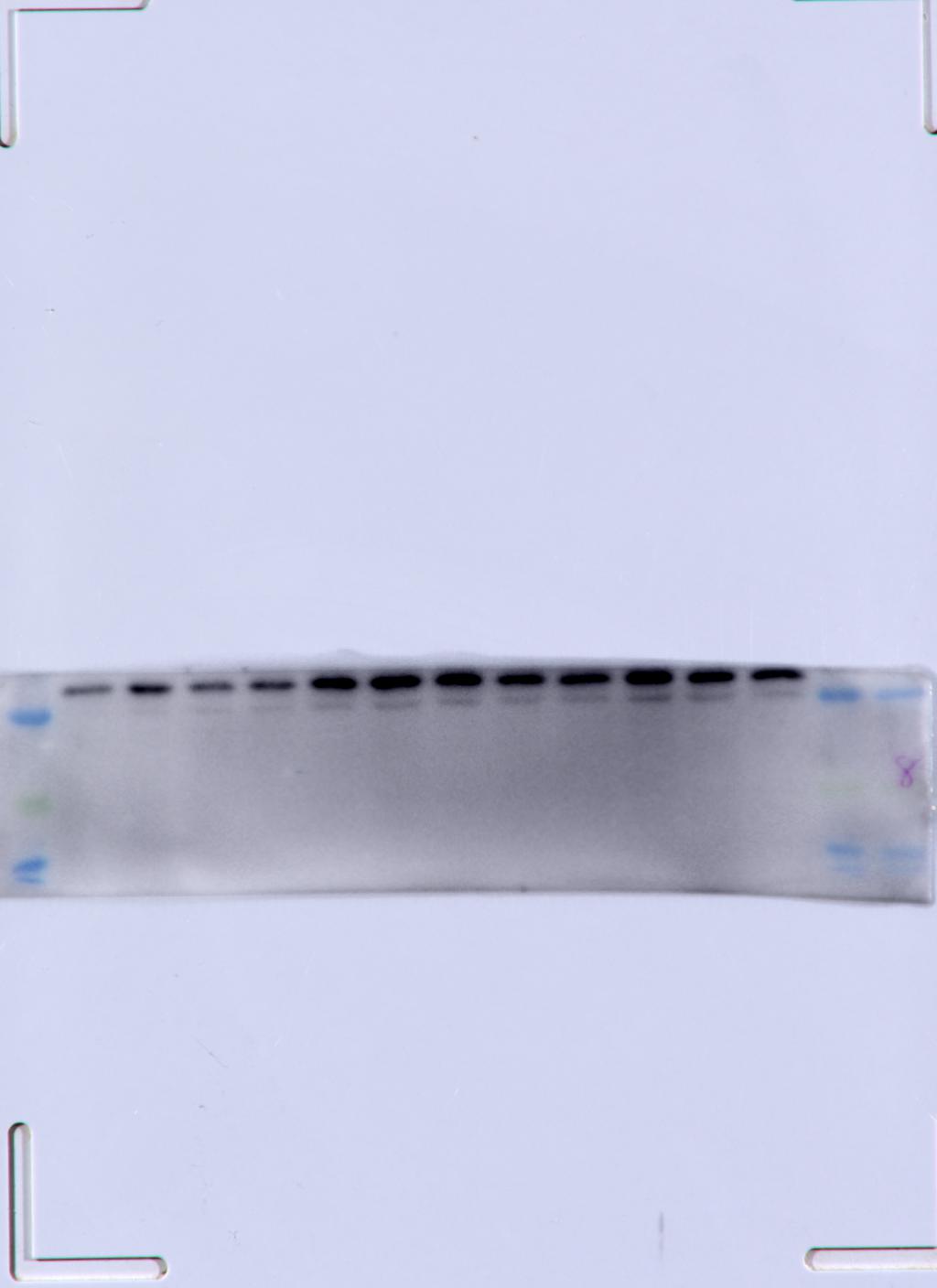

Supplement: Supplementary file 1 [file cancers-14-05188-s001.zip › WB(STING)/figure5B sting.jpg]
